# Excessive Switching in OCD and Paranoia Arises From Different Deficits in Belief-Updating

**DOI:** 10.5334/cpsy.136

**Published:** 2026-06-24

**Authors:** Charlotte M. Freeland, Praveen Suthaharan, Santiago Castiello de Obeso, Christopher Pittenger, Philip R. Corlett

**Affiliations:** 1 Department of Psychiatry, Connecticut Mental Health Center, Yale School of Medicine, New Haven, CT, USA; 2 Wu Tsai Institute, Yale University, New Haven, CT, USA; 3 Department of Psychology, Yale University, New Haven, CT, USA; 4 Center for Brain and Mind Health, Yale University, New Haven, CT, USA; 5 Child Study Center, Yale School of Medicine, New Haven, CT, USA; 6 Department of Neuroscience, Yale School of Medicine, New Haven, CT, USA

**Keywords:** obsessions, paranoia, reversal learning, belief-updating, Bayesian Gaussian graphical modeling

## Abstract

Both obsessions and paranoia are characterized by cognitive inflexibility, particularly in uncertain environments. Differential diagnosis can be challenging and depends on clinical interviews and self-report symptom questionnaires. We predicted that obsessions and paranoia would be associated with distinct suboptimal choice behavioral patterns in our well-established probabilistic reversal learning (PRL) task. Probabilistic reversal learning involves updating beliefs about rewards when contingencies change. Obsessions and paranoia have been linked to excessive switching behaviors during reversal learning; other reports have found perseveration in both OCD and schizophrenia. Here, we analyze data collected from the general population to assess associations between obsessions, paranoia, and PRL task performance. Using a recently developed computational approach – Bayesian Gaussian graphical modeling combined with a Hierarchical Gaussian Filter – we distinguish the impacts of paranoia and obsessions on reversal learning, which we find to be distinct despite the significant correlation of these clinical states. We find that win-switch behavior arises in paranoia from deficits in updating beliefs about uncertainty (i.e., volatility) in the global task structure, whereas excessive switching in OCD arises from diminished belief updating about choice-outcome associations.

## Introduction

Obsessions – recurrent and uncontrollable thoughts with inappropriate content – are central to the diagnosis of obsessive-compulsive disorder (OCD). Similar phenomena are also common in schizophrenia spectrum disorders (SZ) (Grover, Sahoo & Surendran, 2019), occurring in approximately 30% of patients (Swets et al., 2014; Cunill et al., 2023). Furthermore, being diagnosed with OCD is associated with an increased risk for a subsequent schizophrenia diagnosis (Cheng et al., 2019). The differential diagnosis of schizophrenia versus OCD can be challenging, particularly in first-contact patients (Poletti & Raballo, 2019). Comorbid schizotypal symptoms, which are common in OCD (Sobin et al., 2000; West et al., 2025), predict poor responses to standard OCD treatments (Poyurovsky & Koran, 2005). Indeed, treatment-resistant OCD is one of few neuropsychiatric disorders for which invasive neurosurgery is approved as a last resort treatment (De Jesus et al., 2025).

SZ and OCD are both characterized by deficits in cognitive flexibility (Gruner & Pittenger, 2017; Sheffield et al., 2022). Inflexibility can present as fixed, rigid beliefs (i.e., delusions) and/or rumination (e.g., paranoia) in people with SZ and as repetitive, obsessive thoughts and compulsive behaviors in people with OCD. For example, compulsions frequently manifest as ‘checking’ or other repetitive routines to resolve uncertainty about the state of the world (Corlett & Fletcher, 2021; Chamberlain et al., 2021). Adaptive learning requires cognitive flexibility mechanisms that can discriminate inconsequential expected variability (‘expected uncertainty’) from signals of environmental volatility (‘unexpected uncertainty’) (Soltani & Izquierdo, 2019). The computation of stimulus-action-outcome associations and the updating of expected values (prediction errors, PEs) depend on integrated processing across multiple brain regions and hierarchical levels (Collins & Shenhav, 2022); as such, deficits in these cognitive processes can manifest in various clinical presentations, often associated with distress.

Several cognitive models link challenges with regulating uncertainty to dysfunctional beliefs in individuals with OCD (Mcfall & Wollersheim, 1979; Jacoby & Abramowitz, 2017; Strauss et al., 2020). People with OCD tend to overestimate the likelihood of catastrophic events (e.g., the end of the world) (Carr, 1974; Salkovskis, 1989; Tallis, 1995). They also often have unusual beliefs about the controllability of thoughts and the need for mental self-control. The inability to reduce or resolve uncertainty ultimately perpetuates these irrational thoughts and behaviors (Foa & Kozak, 1986). Despite the presence of cognitive inflexibility in both OCD and SZ, the phenomenology of these two conditions diverges, suggesting differences in the underlying neural mechanisms.

A changing environment requires beliefs that are robust to noise yet sensitive to actual change. To investigate the latent cognitive processes underlying cognitive inflexibility, we can turn to behavioral tasks specifically designed to model the dynamics of flexible belief-updating under conditions of uncertainty (Soltani & Izquierdo, 2019). Probabilistic reversal learning (PRL) tasks present subjects with 3 choices, each with two possible outcomes (i.e., reward, no reward) across successive trials. The task is probabilistic because each choice has a different probability of reward (i.e., choice contingency). Choice contingencies change during the task – hence, “reversal” – so participants must form a set of beliefs that are robust enough to probabilistic noise (choice contingencies), yet flexible enough to track actual changes in contingencies (reversals) to maximize the number of rewards earned. On each trial, participants select a choice, the outcome (win or lose) is revealed. The next trial begins when the next choice is presented. This produces four possible selection strategies on the subsequent trial: win-stay (repeat a choice that was rewarded), win-switch (select a different choice following a reward choice), lose-stay (repeat a choice that was not rewarded), or lose-switch (select a different choice following an unrewarded choice). When win-switch or lose-stay strategies are applied excessively, and throughout the duration of the task, these behaviors can be indicative of impaired response selection strategies and underlying neurocognitive deficits (Kalhan et al., 2026). For example, excessive win-switching may be a marker of oversampling, heighted sensitivity to sensory input, reduced attention, or overestimation of transition uncertainty. Conversely, lose-stay behavior becomes perseverative if sustained and may reflect an insensitivity to actual change or low confidence in one’s choices thereby causing their repetition (Fradkin et al., 2018; Robbins, Vaghi & Banca, 2019).

Impaired reversal learning has been reported in various neuropsychiatric disorders that involve frontostriatal circuits, including psychosis, OCD, bipolar disorder, and addictive disorders (Chamberlain et al., 2008). For example, people with schizophrenia perseverate on the Wisconsin card sorting task, although this behavior does not relate to delusions but rather to negative symptoms (Crider, 1997). Paranoia, in people with and without a SZ diagnosis, is associated with increased win-switching, decreased lose-staying, and elevated beliefs about the volatility of the task (Reed et al., 2020; Suthaharan et al., 2021; Sheffield et al., 2022; Rossi-Goldthorpe et al., 2024). Obsessions may be related to perseverative (lose-stay) behavior (Fradkin et al., 2020; Pinciotti, Riemann & Abramowitz, 2021; Rasmussen & Parnas, 2022). It is worth noting that win-shifting may not be specific to paranoia (Fradkin et al., 2020; Suthaharan et al., 2021); Fradkin et al. (2018, 2020) found similar erratic switching in OCD (with the caveat that the number of options available and the underlying changes in contingencies are critical to the behavioral patterns observed). Apergis-Schoute et al. (2023) hypothesized that patients with OCD are impaired in their ability to mediate transitions from one situation/state to another. For example, these individuals commit perseverative errors in some situations (i.e., deterministic tasks with certain rules) but shifting errors in other contexts (i.e., probabilistic tasks with uncertain, probabilistic contingency changes).

In the present study, we combined traditional behavioral models (i.e., win-switch, lose-stay) with Bayesian models to investigate the dynamic updating of priors and posterior beliefs and the latent processes that underlie impairments in reversal learning. We hypothesized that clinically significant paranoia and OCD would predict impairments in probabilistic reversal learning. We, and others, have found that excessive switching in people with paranoia arises from less flexible belief updating in response to volatility (Reed et al., 2020; Suthaharan et al., 2021; Sheffield et al., 2022; Rossi-Goldthorpe et al., 2024). If positive PEs reinforce recent choices and negative PEs punish them (Sakai et al., 2022), we hypothesized that individuals with OCD would have exaggerated negative PEs during the PRL task, that would manifest in decreased lose-stay and increased win-switch behavior. We then explored if excessive switching in OCD arises from an imbalance in negative and positive PEs and reduced belief-updating about choice contingencies or task volatility.

To describe such volatility beliefs quantitatively, we can fit Bayesian inference (BI) models to participants’ PRL behavior to estimate computational parameter values that account for individual choice patterns. Bayesian inference is versatile and has been applied across scientific disciplines, including computational psychiatry (Hess et al., 2025). Whereas traditional reinforcement learning models have been applied to choice behavior in PRL tasks, BI models are better equipped to capture dynamic trial-by-trial choices and sensory inputs from choice outcomes, generating predication errors that update an individual’s probabilistic model, enabling behavioral adaptions in response to task volatility (Bottemanne, 2025). One such Bayesian model, the Hierarchal Gaussian Filter (HGF) model, is designed to capture hierarchal belief updating and volatility beliefs (see Methods for further details) (Mathys et al., 2014). HGF modeling has been applied to choice behavior in PRL tasks in human participants, non-human primates, and rodents to infer latent processes that lead to distinct behavioral strategies. For example, win-switch behavior has consistently correlated with paranoid beliefs and HGF volatility estimates, across numerous replicated studies (Reed et al., 2020; Suthaharan et al., 2021; Sheffield et al., 2022; Rossi-Goldthorpe et al., 2024; Suthaharan et al., 2024; Suthaharan et al., 2026).

We aimed to establish whether there is a model-based explanation for reduced perseveration (lose-stay) or excessive switching (win-switch) in participants with self-reported OCD symptoms. We then compared previously reported HGF parameter estimates (Suthaharan et al., 2021) in participants with clinically significant paranoia and/or OCD to those in participants with low symptom scores and performed network analyses (Bayesian Gaussian graphical modeling). Together, these computational methods may provide insight to latent cognitive mechanisms that underlie, and distinguish, OCD and paranoia.

## Methods

This was a post hoc observational study that analyzed data collected and reported by Suthaharan et al. (2021) and publicly available at https://github.com/psuthaharan/covid19paranoia. Their data were collected at the Connecticut Mental Health Center in accordance with guidelines from Yale University’s Human Investigation Committee, which provided ethical review and exemption approval (no. 2000026290). Written informed consent was provided by all research participants. Questionnaires were administered via the Qualtrics® survey platform (Qualtrics Labs, Inc., Provo, UT). Behavioral data were collected using custom task code on the online research platform Amazon Mechanical Turk Human Intelligence, with a total of 1,010 participants recruited online via CloudResearch (Litman, Robinson & Abberbock, 2017). The analysis presented in this manuscript includes a sample of 403 of these participants, who completed both the paranoia (R-GPTS) and OCD (DOCS) self-report questionnaires. These data were gathered between March 16th and June 31st, 2020 (Suthaharan et al., 2021).

### Clinical Assessments and Sample Characteristics

Participants completed several self-report questionnaires, including the revised Green et al. Paranoid Thoughts Scale (R-GPTS) (Freeman et al., 2021), Dimensional Obsessive-Compulsive Scale (DOCS) (Abramowitz et al., 2010), Beck’s Anxiety Inventory (BAI) (Beck et al., 1988), and Beck’s Depression Inventory (BDI) (Beck et al., 1961). Demographic information (age, sex, ethnicity, etc.) was also collected (Suthaharan et al., 2021).

Of the 403 participants included in the present study, 154 met the clinical cut-off for high paranoia using the R-GPTS (score ≥ 11 out of a maximum of 80), and the remaining 249 participants were classified as sub-clinical or “low” paranoia (Reed et al., 2020; Suthaharan et al. 2021; Rossi-Goldthorpe et al., 2024). 170 participants were classified as individuals with high OCD and 233 participants with low OCD, based on a clinical cut-off of adjusted DOCS scores (score > 13 out of a maximum of 60, see below). These groupings are not distinct, as there is overlap between participants assigned to paranoia and OCD groups.

### Impact of COVID-19 Pandemic on OCD Symptoms

OCD severity in this study was assessed with the DOCS questionnaire, which includes 20 items across four broad symptom domains: (1) germs and contamination; (2) responsibility for harm, injury, or bad luck; (3) unacceptable obsessional thoughts; and (4) symmetry, completeness, and exactness (Abramowitz et al., 2010). A limitation of this study is that the dataset analyzed was collected from mid-March to mid-July 2020, during the onset and peak of the COVID-19 pandemic, and thus, requiring consideration of the pandemic’s potential impact on OCD symptoms. Indeed, a study of 829 non-clinical U.S. participants reported a median increase in DOCS scores from 6 (pre-pandemic) to 16 (July 2020, mid-pandemic), indicating symptom worsening (Fontenelle et al., 2021). Similarly, a systematic review of individuals with pre-existing clinical OCD found most studies reported heightened symptoms, particularly in those with contamination-related OCD, during the early pandemic (Guzick et al., 2021). Given these findings and the timing of data collection, we chose to exclude DOCS items related to germs and contamination from participants’ total DOCS scores.

### Probabilistic Reversal Learning Task

**Figure 1:**
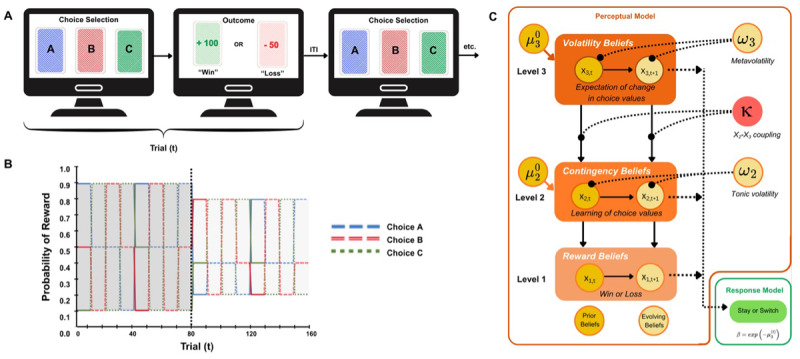
**(A)** Depiction of the 3-choice probabilistic reversal learning task (3-PRLT) and **(B)** reward contingency schedule. **(C)** Hierarchal Gaussian Filter (HGF) computational modeling of 3-choice PRL task behavior to capture belief updating in an uncertain and volatile task environment.

Participants completed a 3-option probabilistic reversal learning task (3-PRLT) (Reed et al., 2020; Suthaharan et al., 2021; Sheffield et al., 2022; Rossi-Goldthorpe et al., 2024; Suthaharan et al., 2024) wherein they are presented with three decks of cards, each with winning and losing cards, but some with more winning cards than others. Their goal is to find and select the best deck to maximize points. They are also informed that the best deck may change during the task. Upon selection of a deck on each turn, the participant receives feedback, either a win of 100 points or a loss of 50 points (Figure 1A). To ensure comprehension of task instructions and objective, participants complete 6 practice trials prior to advancing to the task start. The 3-PRLT is comprised of 4 blocks with 40 trials each (160 total trials). In the first two blocks, the three decks are assigned reward probabilities of 90%, 50% and 10% respectively (i.e., one deck will win on 9 out of 10 turns, one will win on 5 out of 10 turns, and one will win on 1 out of 10 turns). After a participant selects the highest probability deck on 9 out of 10 consecutive trials, the reward probability assignments switch between the three decks; unexpected uncertainty is the perceived likelihood that such a change has occurred. In blocks 3 and 4 of the task, we increase the level of unexpected uncertainty by changing the reward probability contingencies to 80%, 40% and 20%. This volatile task design requires participants to dynamically track and update beliefs about 1) choice contingencies (‘expected uncertainty’) and 2) changes in the underlying choice contingencies themselves (‘unexpected uncertainty’).

Values for win-switch and loss-stay rates were quantified for each participant (as reported in Suthaharan et al., 2021) to model participants’ belief updating in response to uncertain reward contingencies in a volatile task environment. For a given trial (*t*), a participant selects one of three possible choices *X_t_* ∈ {*A, B, C*}, with each choice yielding one of two possible outcomes *Y_t_* ∈ {0, 1} (win or loss). On the subsequent trial (*t* + 1), participants can either make a different choice (*X*_*t* + 1_ ≠ *X_t_*) or repeat their prior choice (*X*_*t* + 1_ = *X_t_*). For each participant, we calculated win-switch rate (WSR) as the probability of selecting a different choice following a win outcome and lose-stay rate (LSR) as the probability of selecting the same choice following a loss outcome across 160 trials.


$$\begin{array}{l} \text{WSR}(x)=P(X_{t}=x,Y_{t}=1,X_{t+1}\neq X_{t})\\ \text{LSR}(x)=P(X_{t}=x,Y_{t}=0,X_{t+1}=X_{t}) \end{array}$$

In this 3-arm PRL task, win-switch and lose-stay rates were both negatively correlated with total points earned (Supplementary Figure 1), suggesting that, in this probabilistic task design with a finite number of choices, win-switching and lose-staying are suboptimal choice strategies.

Excessive win-switching has been seen across the continuum of paranoia and in patients with schizophrenia, with computational modeling revealing this behavior to be driven by strong prior beliefs about task volatility a well as poor learning about real volatility (Reed at al., 2020; Suthaharan et al., 2021; Rossi-Goldthorpe et al., 2024). However, whether excessive switching across the continuum of OCD arises from the same or different latent belief-updating processes has yet to be explored.

### Computational Modeling of PRL Behavior with Hierarchal Gaussian Filter

Probabilistic reversal learning involves decision-making under uncertainty about which option to choose and whether the options have recently changed or even reversed in their value. Computational modeling of task performance under uncertain and volatile conditions provides access to estimates of latent decision-making processes. We modeled beliefs using the Hierarchal Gaussian Filter (HGF), a Bayesian model for perception, learning, and action. It comprises a perceptual model to capture participants’ task beliefs and a response model that governs how beliefs are converted into choices (Mathys et al., 2014). ‘Hierarchical’ refers to multiple layers of belief representation (i.e., beliefs about reward contingencies stimuli and beliefs about how those contingencies might change over time). Gaussian refers to how the states in the model evolve over time as Gaussian random walks, with each walk’s step size determined by the next highest level of the hierarchy, that is, each belief layer updates the layer below considering evolving experiences. ‘Filter’ comes from the fact that we are trying to estimate states on a given trial, based on the previous trial mean and variance. The hierarchal layers interact and influence one another through learning rate parameters (Figure 1C).

In this HGF model (Reed et al., 2020; Suthaharan et al., 2021), level 1 (x_1_) characterizes trial-by-trial perception of task feedback (e.g., win or loss), representing an individual’s reward beliefs. Level 2 (x_2_) captures choice-outcome associations, forming an individuals’ contingency beliefs, where estimates of parameter *µ^0^_2_* captures prior beliefs about choice-outcome associations, and with successive trials, *ω_2_* captures the tonic volatility of choice-outcome associations. Lower *ω_2_* indicates that subjects are slower to update their beliefs about the value of each option, and hence they main rigid beliefs about the underlying choice contingencies. Phasic coupling between levels 2 and 3, parameter k, approximates the influence of unexpected uncertainty – that is, the effect of volatility perceptions on learning about choice contingencies. Higher k implies participants are more likely to perceive volatility and hence demonstrate faster updating of choice contingencies. Level 3 (x_3_) renders beliefs about volatility in the overall task environment (i.e., the likelihood of the choice-outcome associations to change over time). *µ^0^_3_* estimates prior beliefs about volatility, and with successive trials, perceptions about expectations of volatility, or meta-volatility, is captured by the parameter *ω_3_*. The lower *ω_3_*, the slower a subject is to adjust their volatility belief; they adhere more rigidly to their volatility prior (*µ^0^_3_*).

HGF models have previously been applied to participants’ 3-PRL choices (Reed at al., 2020; Suthaharan et al., 2021; Rossi-Goldthorpe et al., 2024; Suthaharan et al., 2024) allowing for inference of participants’ initial beliefs (i.e., priors) about task volatility, their readiness to learn about changes in the task volatility itself (meta-volatility) and learning rates that captured their expected and unexpected uncertainty regarding the task. A three-level perceptual model with a softmax decision model (likelihood of exploration vs exploitation) dependent upon third level volatility and inverted the model from subject data (trial-by-trial choices and feedback) to estimate parameters for each participant. Parameter recovery was conducted by correlating true and recovered parameter estimates across participants. Recovery performance was averaged across 10 independent simulations. Recovery was strong for four of the five parameters (*r* ≥ 0.74) and moderate for *ω_3_* (*r* = 0.45), consistent with known differences in parameter identifiability in hierarchal Bayesian models such as the HGF (Supplementary Figure 9) (Reed at al., 2020; Suthaharan et al., 2021; Suthaharan et al., 2026). Overall, these results support model identifiability for group-level inference under this 3-level HGF configuration.

### Bayesian Gaussian Graphical Modeling of Symptoms & PRL Behavior

It is often assumed that symptoms are manifestations of some common underlying neurobiological factor. Network analyses, in contrast, conceptualizes disorders as systems of causally connected symptoms, rather than as effects of a unitary latent dysfunction (Borsboom & Cramer, 2013). In this framework, symptom-symptom relationships can provide empirical evidence for covariance between symptoms, which then may inform a model of their role in the etiology of a disorder.

Partial correlation networks, such as Bayesian Gaussian graphical models (BGGMs), have become increasingly popular in psychological and behavioral science for studying the conditional (in)dependencies between variables (Williams & Mulder, 2019; Williams et al., 2020; Rossi-Goldthorpe et al., 2024). For example, items in a psychometric scale are designed to measure a dynamic behavioral system that influence and interact with each other and cannot be assumed to be independent variables, as is assumed in traditional structural equation models. Bayesian Gaussian graphical models (BGGMs) capture the conditional dependency structure among a set of multivariate variables, while controlling for the effects of partial correlations amongst all other variables in the model. This could be understood as a set of many Bayesian multiple regressions. For example, if the edge between nodes A and B is the estimate of a regression to predict A with B by controlling for all other variables (C and D), then the edge between B and C is the regression predicting B with C and controlling by A and D, etc. The presence of an edge between two nodes indicates that the two variables are conditionally dependent (i.e., the 95% credible interval of their dependence does not include the zero). A green edge represents a positive partial correlation and a red edge a negative partial correlation. A limitation of BGGMs is that the number of variables included in the model cannot be greater than the number of observations (Williams & Mulder, 2019).

BGGMs are organized around two central approaches: (1) estimation, which estimates the uncertainty of the posterior distribution for each partial correlation, and 2) hypothesis testing, which used Bayes factor methods to determine the entire probability distribution of parameters and thereby incorporates model uncertainty (Williams et al., 2020). The R package ‘BGGM’ provides tools for confirmatory testing and comparison of graphical models, thereby serving as both a statistical and data visualization approach (Williams & Mulder, 2019). For hypothesis testing, we used the Bayes’ factor-based method explore to learn about the conditional (in)dependence structure, or evidence for the null hypothesis (i.e., participants with paranoia and OCD perform similarly on the PRL task). The estimate function with the option alternative = “exhaustive” compared the posterior hypothesis probabilities for a positive (ρ > 0 [*P*(H1)]), negative (ρ < 0 [*P*(H2)]), or null (ρ = 0 [*P*(H0)]) relation to the alternative hypothesis. We then computed the posterior mean, standard deviation, and posterior hypothesis probabilities for each relation, or pair of nodes, within each network.

In the present study, we leveraged BGGM to examine the conditional (in)dependence between self-reported symptoms (paranoia and obsessions) and PRL task behavior. Continuous values were used for paranoia and OCD rather than low/high groups, allowing for comparison across the spectrum of symptom severity and within relationship to each other.

### Statistics

To test the prediction that win-switch or lose-stay behavior is a function of paranoia and/or OCD severity, we fit generalized linear models using the R package *lme4* (Bates et al., 2015). Shapiro-Wilk tests were used to check normality assumptions, Levene’s test were used to check homogeneity assumptions, and variance inflation factors were computed to check multicollinearity assumptions. The predictor variables ‘paranoia’ and ‘OCD’ showed non-normal, left-skewed distributions and, heteroscedasticity, and they were significantly correlated with each other (VIF > 5; see Supplementary Figure 2). The response variables win-switch rate (WSR) and lose-stay rate (LSR) are continuous probabilities bounded between 0 and 1, with left-skewed distributions. To flexibly model these response variables, our models assumed a quasibinomial error distribution which can more appropriately handle unbalanced proportion data without the assumption of a fixed ratio of residual deviance to residual degrees of freedom (McCullagh & Nelder, 1989; Hector, 2015).

We then tested the hypothesis that the latent computational processes underlying choice behavior (captured by HGF model parameters) predicted paranoia and/or OCD symptom severity. Model parameters (*µ^0^_2_, µ^0^_3_, ω_2_, ω_3_*) were continuous and approximately normally distributed, based on residual diagnostics (see Supplementary Figure 7A–E). We therefore modeled symptom severity using generalized linear models with a Gaussian distribution and identity link function. This specification is equivalent to linear regression and was chosen to allow consistency with the broader modeling framework while accommodating continuous outcomes without transformation. HGF model parameter k had positive, non-normally distributed, continuous values, so we adopted a generalized linear model with a quasibinomial error distribution. Outliers for this parameter were identified using a robust criterion based on the median absolute deviation (MAD) (Arachchige & Prendergast, 2026). Two observations exceeded ±3 robust standard deviations from the median (z* ≥ 5.4) and were excluded from subsequent analyses.

To model interaction effects between continuous predictors and outcome variables, we computed estimated marginal means using the *emmeans* package (Lenth, 2024), with Tukey adjustment methods to account for multiplicity in post hoc contrasts. Post hoc testing of paranoia and OCD group differences on the effects of demographic and task performance measures were assessed with Kruskal-Wallis and Wilcoxon t-tests. Spearman’s correlations were computed with Pearson’s rho.

Different analyses addressed distinct inferential levels, and statistical frameworks were selected according to the research question. Frequentist generalized linear and mixed-effects models were used for confirmatory parameter-level inference in hierarchically structured behavioral data, whereas Bayesian Gaussian graphical models were used to characterize conditional dependence structure and quantify uncertainty at the network level.

All analyses were performed in R Studio v.4.2.764. All general data cleaning and figure generation were performed in the *Tidyverse* ecosystem of R packages (Wickham et al., 2019). Manuscript figures were compiled in Adobe Illustrator (v.29.7.1). Figure 1 was adapted from Suthaharan et al. (2021) and modified in Canva. All code, figures, and model comparisons are publicly available at https://github.com/CharlotteFree/excessiveSwitching.

## Results

### PRL Task Behavior & Clinical Symptoms

**Figure 2:**
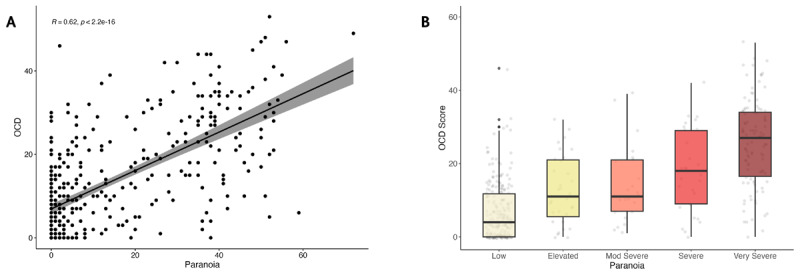
**(A)** OCD symptom scores had a strong positive correlation with paranoia symptom scores. **(B)** The clinical cutoff used to delineate low from high paranoia groups (R-GPTS > 11) includes individuals with elevated, moderately severe, and very severe R-GPTS scores.

Obsessions and paranoia often develop under conditions of uncertainty. Both entail rigid and persistent patterns of thought. As expected in our sample from the general population, OCD symptom scores were positively correlated with paranoia symptom scores (Figure 2A; *r* = 0.63; Supplementary Figure 2) and increased across the clinical spectrum of paranoia (Figure 2B; *p* < 0.001).) There was no significant effect of participants’ sex on paranoia or OCD symptom scores (Supplementary Figure 3A). Participants in the high paranoia group were significantly younger than those with low paranoia scores (*p* < 0.001), although there was no effect of age on OCD group or self-reported symptom scores (Supplementary Figure 3B).

**Figure 3:**
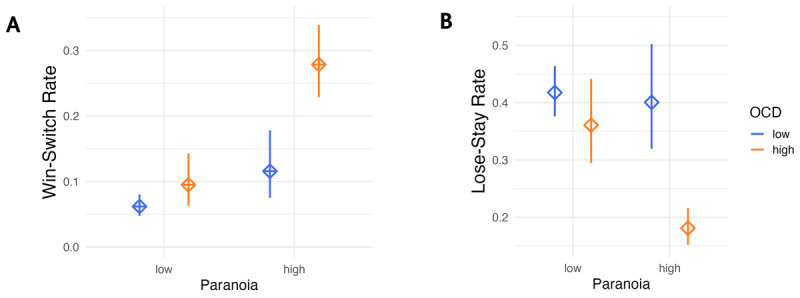
Estimated marginal means of **(A)** win-switch and **(B)** lose-stay rates by low/high paranoia group (x-axis) and low/high OCD group (blue/orange). Error bars show the upper and lower 95% confidence interval. **(A)** Participants with high paranoia and high OCD scores had significantly greater rates of win-switching, compared to those with either low paranoia and/or low OCD. **(B)** Participants with high paranoia and high OCD symptom scores had significantly lower rates of lose-staying, compared to those with either low paranoia and/or low OCD.

We observed a greater rate of win-switching in participants with high paranoia, compared to those with low paranoia scores (*β* = 0.63, *t*_(794)_ = 2.43, *p* = 0.015), consistent with past results [31,32.5]. We observed a similar pattern of excessive win-switching in participants in the high OCD symptom group (*β* = 0.43, *t*_(794)_ = 1.73, *p* = 0.084), but no significant interaction between paranoia and OCD groups (Figure 3A; Table S1a). Post hoc pairwise comparisons using estimated marginal means with a Tukey adjustment revealed that subjects with high paranoia and high OCD win-switched 22% more often those with low paranoia and low OCD (*p* < 0.001). These participants with high paranoia-high OCD also win-switched significantly more than participants with high paranoia-low OCD and low paranoia-high OCD (*p* = 0.002 and *p* < 0.001, respectively; Supplementary Table S1c). These findings replicate prior work showing paranoia is associated with increase win-switching and newly describe a similar pattern of excessive switching in participants with clinically significant OCD symptoms.

Next, we examined choice perseveration after unreinforced choices (losses). While there was no significant effect of paranoia group or OCD group on lose-stay rates, there was a significant interaction between paranoia and OCD (*β* = –0.65, *t*_(794)_ = –3.48, *p* = 0.001; Table S2a). Participants with high paranoia and high OCD had a 2.3-fold reduction in lose-stay behavior, compared to those with low paranoia and low OCD (*p* < 0.001; Figure 3B). These high paranoia-high OCD participants also had significantly lower rates of lose-staying than those with high paranoia-low OCD and low paranoia-high OCD (*p* < 0.001 and *p* < 0.001, respectively; Table S2c). Thus, aligning with our hypotheses, participants with *both* high paranoia and high OCD demonstrated greater win-switching and reduced lose-staying, than participants in the low groups.

Given that both win-switching and lose-staying are suboptimal choice strategies (Supplementary Figure 1), we tested whether other metrics of PRL performance (i.e., total score, decision/choice latency) were associated with symptom severity. There was no significant effect of paranoia group, OCD group, or an interaction between paranoia and OCD groups on total points earned (Supplementary Figure 5; Table S3a). Post hoc pairwise comparisons revealed that only subjects with high paranoia and high OCD earned significantly fewer points than participants with low paranoia and low OCD (Table S3b). For choice latency, there was a significant effect of paranoia group (*β* = 74.90, *t*_(794)_ = 2.04, *p* = 0.041) and a significant interaction between paranoia and OCD groups (*β* = 191.04, *t*_(794)_ = 3.36, *p* < 0.001) (Table S4a). Post hoc comparisons of estimated marginal means revealed increased choice latency is driven by high paranoia, regardless of low/high OCD group (Table S4b). Future research could explore how choice latency and cognitive processing load interact with the latent processes (or beliefs) that guide choice selection.

**Figure 4:**
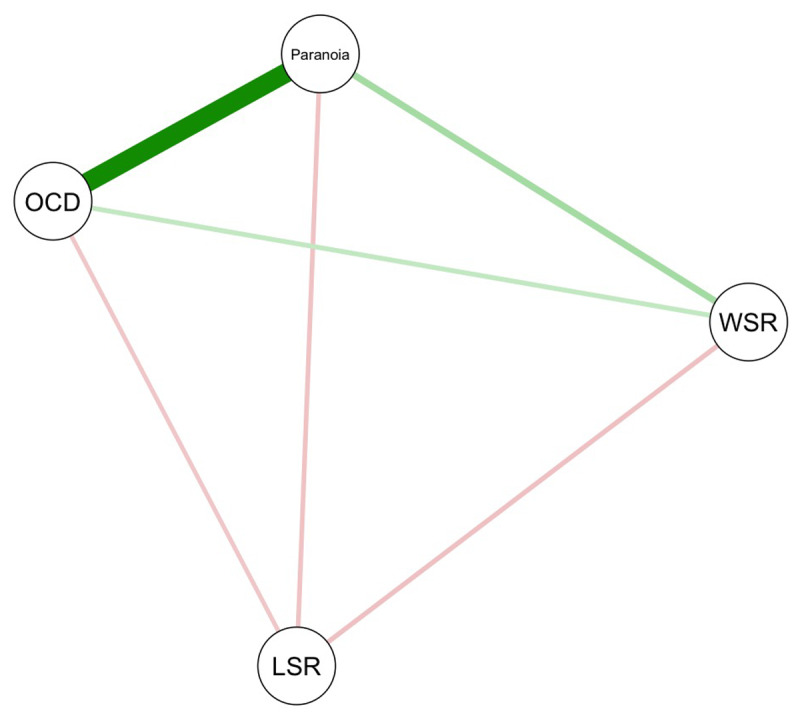
Bayesian Gaussian graphical model (BGGM) of the relationships between paranoia, OCD, win-switch rate (WSR), and lose-stay rate (LSR). A green edge represents a positive partial correlation and a red edge a negative partial correlation.

We subsequently applied a BGGM approach to determine if these between-group relationships with win-switch and loss-stay behavior exist across the continuum of paranoia and OCD symptom scores. BGGM revealed that the strength of the associations between symptoms and win-switch and lose-stay behavior is not equivalent. Win-switch rate has a strong positive association with paranoia [*P*(H1) = 1.0] and OCD [*P*(H1) = 0.806]. Lose-stay rate had a weaker negative association with both paranoia and OCD symptoms [*P*(H2) = 0.707 and *P*(H2) = 0.549, respectively] (Figure 4). In summary, along the spectrum of paranoia and OCD scores, paranoia and OCD have strong positive association with each other [*P*(H1) = 1.0] and with win-switching, and weaker negative associations with lose-staying (Table S5).

### Hierarchal Gaussian Filter (HGF) Model of PRL Behavior

**Figure 5:**
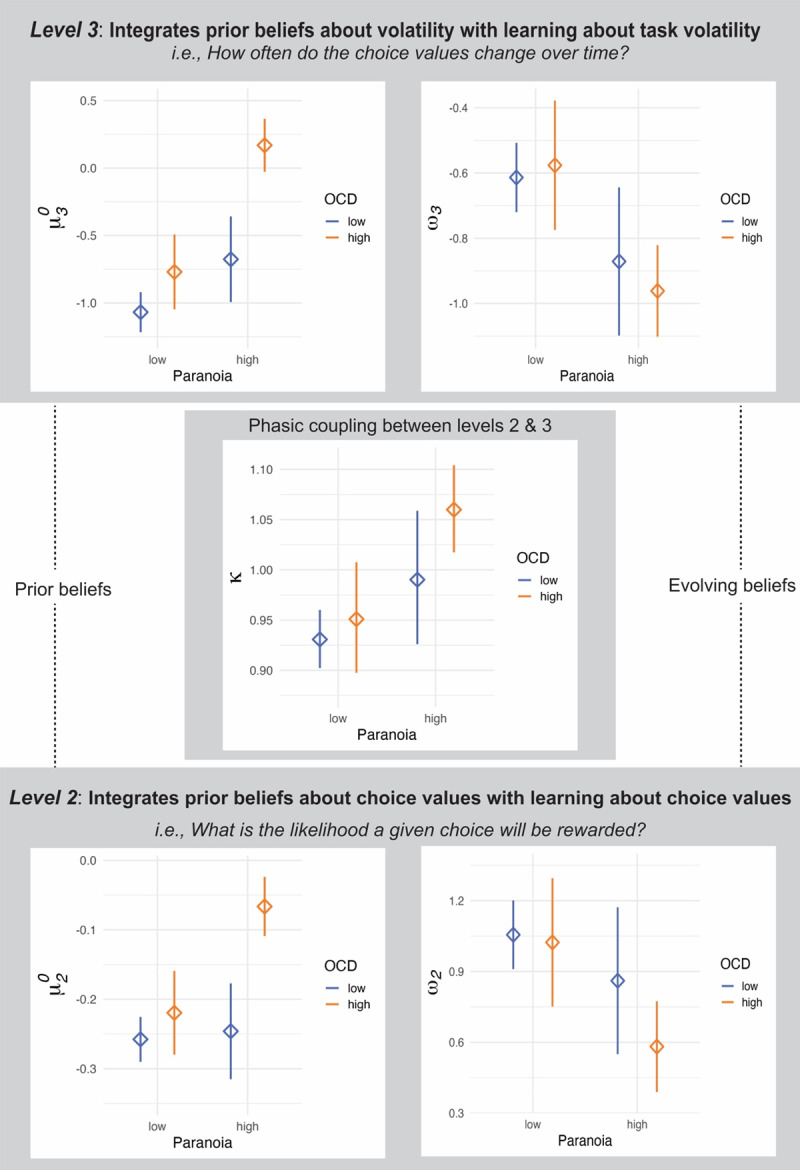
HGF model parameters estimates (derived from PRL task behavior) comparing participants with low and high paranoia (x-axis) and low (blue) and high (orange) OCD.

We modeled HGF model parameters previously reported for this dataset [33] to investigate whether the excessive win-switching seen in individuals with high paranoia and/or high OCD arise from similar latent cognitive processing mechanisms. Prior contingency beliefs, *µ^0^_2_*, were not predicted by paranoia group or OCD group, but there was a significant interaction between paranoia and OCD groups (*β* = 0.14, *t*_(794)_ = 2.62, *p* = 0.009; Table S6a). Post hoc comparisons revealed that participants in both high paranoia and high OCD groups had significantly stronger prior beliefs about reward contingencies than those with either low paranoia or low OCD (*p* < 0.001; Figure 5, bottom left; Table S6c). Evolving beliefs about choice contingencies, captured by *ω_2_*, were not predicted by paranoia group, OCD group, or interaction between groups (Table S7a); however, in post-hoc pairwise comparisons, participants with high paranoia and high OCD had significantly less updating of contingency beliefs than those in the low paranoia group, regardless accompanying low/high OCD (*p* = 0.001, *p* = 0.046, respectively; Figure 5, bottom right; Table S7c).

In addition to updating beliefs about choice contingencies, participants must also learn about likelihood that the choice contingencies will change (i.e., volatility of choice contingencies), and how so, to achieve optimal performance (i.e., to score the most points). Phasic coupling between levels 2 (contingency beliefs) and level 3 (volatility beliefs) is captured by the magnitude of *κ* – was not predicted by paranoia group, OCD group, or an interaction between paranoia and OCD groups (Figure 5, middle; Table S8).

At level 3, there was a significant main effect of paranoia group, but not OCD group on prior beliefs about the volatility of choice contingencies (*µ^0^_3_*) (paranoia: *β* = 0.40, *t*_(794)_ = 2.20, *p* = 0.028) and a significant interaction between paranoia and OCD (*β* = 0.55, *t*_(794)_ = 2.20, *p* = 0.028; Table S9a). Post hoc comparisons using estimated marginal means revealed that participants with high paranoia and high OCD had significantly greater *µ^0^_3_* values than participants with either low paranoia and/or low OCD (p < 0.001 for all three comparisons; Table S9c). The magnitude of *ω_3_* (evolution of volatility beliefs) was significantly lower in participants with high paranoia scores, compared to those with low paranoia (*β* = –0.26, *t*_(794)_ = –2.20, *p* = 0.044; Table S10a) but there was no effect of OCD group or interaction. In other words, clinically significant paranoia and OCD symptoms interact to produce high expectations of volatility in choice values, but diminished updating about volatility in choice contingencies is driven solely by high paranoia.

To summarize findings from this hierarchal computational approach, participants with high paranoia and high OCD had stronger prior beliefs about choice-outcome contingencies (*µ^0^_2_*) and their volatility over time (*µ^0^_3_*), compared to participants with either low paranoia or low OCD. However, updating of beliefs about choice contingencies (*ω_2_*) and their volatility (*ω_3_*) was significantly diminished in participants with high paranoia and high OCD, compared to those with low paranoia of any OCD status.

### Bayesian Gaussian Graphical Modeling of Symptoms & PRL Behavior

**Figure 6:**
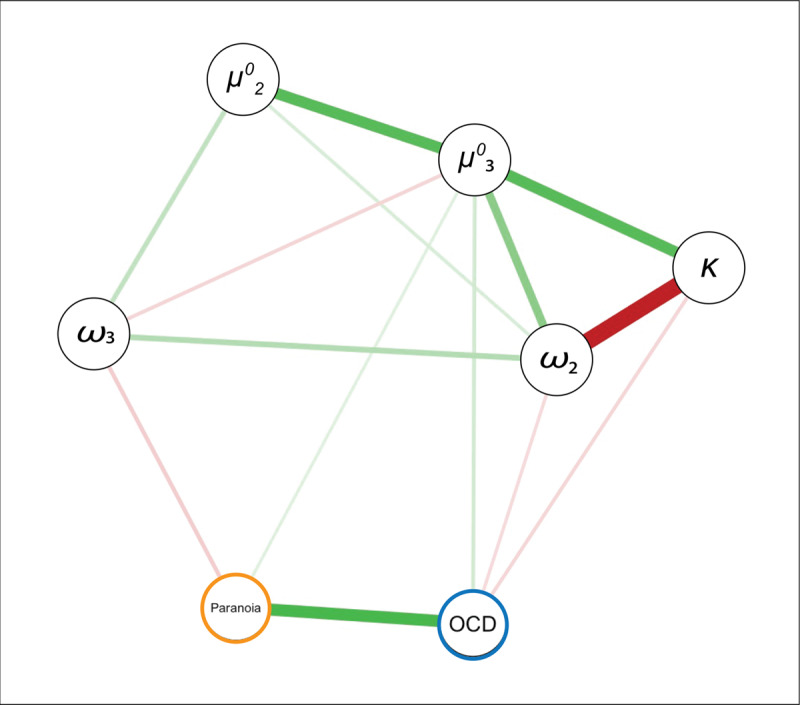
Bayesian Gaussian graphical model (BGGM) of the relationships between paranoia (orange outline), OCD (blue outline), and HGF model parameters. A green edge between nodes represents a positive partial correlation and a red edge a negative partial correlation. Edge thickness represents the strength of the correlation between pairs of nodes, while controlling for the effects of partial correlations amongst all other variables in the model.

We next used Bayesian Gaussian graphical modeling (BGGM) (Williams & Mulder, 2020) to explore the relationship of computational model parameters derived from PRL behavior with symptoms. Of note, these analyses do not divide participants into ‘high’ and ‘low’ paranoia and OCD groups but rather treat both symptoms as continuous variables. Paranoia had no association with evolving contingency beliefs (*ω_2_*) but a strong negative association with evolving volatility beliefs (*ω_3_*) [*ω_2_*: *P*(H0) = 0.964; *ω_3_*: *P*(H2) = 0.999]. Conversely, OCD had a negative association with updating contingency beliefs and no association with evolving beliefs about volatility [*ω_2_*: *P*(H2) = 0.830; *ω_3_*: *P*(H0) = 0.978] (Figure 6; Table S11). Paranoia and OCD were not directly associated with between prior beliefs about choice contingencies (*µ^0^_2_*) but were positively associated with prior beliefs about volatility (*µ^0^_3_*) [paranoia: *P*(H1) = 0.572; OCD: *P*(H1) = 0.988]. Additionally, OCD, but not paranoia, had a negative association with the phasic coupling of reward and volatility beliefs (*κ*) [paranoia: *P*(H0) = 0.837; OCD: *P*(H2) = 0.955]. Importantly, the relationships with these parameters were not confounded by comorbid anxiety and depression symptoms (Supplemental Figure 8; Table S12).

In summary, our results provide evidence to support that deficits in PRL performance (i.e., win-switching) in participants with paranoia are driven by strong prior beliefs about volatility and diminished belief-updating and learning from volatility, whereas suboptimal performance in participants with OCD manifests as strong prior beliefs about choice values and poorer learning about choice contingencies. Reduced belief-updating at level 2 may result in less integration of dynamic level 2 and 3 beliefs, such that individuals with high OCD scores also demonstrate less flexible belief-updating in response to volatility in choice contingencies.

## Discussion

People with paranoia are more likely to switch between choices, even after a correct or “winning” choice, a behavior driven by strong prior beliefs in likelihood of the “deck being shuffled” (volatility) and less belief updating about actual choice contingencies. Individuals with high OCD symptoms also exhibited elevated rates of win-switch behaviour, but these were instead driven by strong prior beliefs about the choice contingencies and less flexible belief-updating in response to volatility in choice contingencies. The qualitative differences between the effects of OCD symptoms and those of paranoia is most clearly shown in the BGGM in Figure 6, which reveals similar relationships to *µ^0^_3_* (i.e., similar a priori beliefs about the stability of the world), but distinct relationships to *ω_3_* (belief updating in response to volatility) and *ω*_2_ (belief updating about choice-outcome associations), when taking both paranoia and OCD symptoms into account.

Parameter recovery procedures indicated that the model is largely identifiable, with four parameters showing strong recovery and *ω*_3_ showing comparatively lower precision (*r* = 0.45). While this is consistent with known limitations in hierarchical Bayesian model inversion (Reed et al., 2020; Rossi-Goldthorpe et al., 2024; Suthaharan et al., 2026), it is worth noting that poorer recovery implies that there is likely a better model of these data and this task. Correlation between parameters might mean the same, implying that the behavioral effects are not uniquely specified. Of course, there are instances when mechanisms trade off – for example, of prediction errors drive attentional allocation, we might want to attend to highly predictable things (Mackintosh, 1975) or highly unpredictable (Pearce & Hall, 1980). Yet, in both cases, prediction error and learning rate are correlated – albeit in different directions.

The considerable correlation between OCD-like experiences and paranoia (Figure 2) has here obscured as clear a dissociation, which BGGM visualization makes clear: there are associations between OCD and reward learning rate that are absent for paranoia (when taking the counterpart symptom into account). This distinction – between abstractions of belief about reward and about change – is mirrored in recent primate computational and behavioral neuroscience data. Macaque monkeys can also learn this 3-option PRL task. In these animals, lesions of the mediodorsal thalamus promote win-switching behavior, whereas lesions of the orbitofrontal cortex impair learning about changes in reward value (Suthaharan et al., 2024). These primate data, together with our observations in humans, suggest that mediodorsal thalamus might be associated with paranoia, as has been observed with lesion network mapping (Loh et al., 2022). Further, these data may shed light on the relationship between OCD and orbitofrontal cortex dysfunction (Ursu & Carter, 2009).

Our findings suggest possible explanations for conflicting findings in previous work examining the PRL in OCD. Some studies have found excessive switching during probabilistic reversal learning in OCD, while others have reported perseveration (Crider, 1977; Rasmussen & Parnas, 2022). The number of response options, the underlying contingencies, and the changes in those contingencies may all influence the win-switching observed (Soltani & Izquierdo, 2019; Sakai et al., 2022). Furthermore, since OCD and paranoia are often comorbid, and strongly (but not perfectly) correlated, it is possible that inconsistencies in PRL behavior in OCD reflect the impact of varying levels of paranoia. We suggest measuring and ideally covarying for paranoia in future investigations of the relationships between OCD and belief updating under uncertainty.

Another important caveat that these experiments were in ostensibly healthy people, free from psychiatric illness. This work bears replication in well characterized samples of patients with OCD and persecutory delusions in the context of schizophrenia. This is a worthwhile exercise since differential diagnosis of schizophrenia versus OCD can be challenging, particularly in first-contact patients (Cheng et al., 2019), and comorbid schizotypal symptoms, which are common in OCD (Poletti & Raballo, 2019), predict poor responses to standard OCD treatments (Sobin et al., 2000). Furthermore, a phenomenological approach to OCD diagnosis allows for obsessions that reach delusional intensity, and thus, admits considerable overlap in the anomalies of experience that signify OCD and schizophrenia. Interestingly, Bleuler remarked in a footnote that “the typical cases [of compulsive neurosis] have so much about them that is schizophrenic in appearance and heredity, that one cannot repress the suspicion, that they are actual schizophrenics whose symptomatology exhausts itself in compulsive syndrome” (West et al., 2025). Whereas classic psychopathology emphasized form rather than content for classification, the DSM-5 text mentions “specific contents as additional features supporting diagnosis [of OCD]”, i.e., cleaning, symmetry, forbidden or taboo thoughts (aggressive, sexual or religious) and fears of harm to oneself and others. However, symmetry, aggressive, violent, or sexual contents have also been regarded as characteristic of schizophrenia (Poyurovsky & Koran, 2005). This is a problem both for categorial diagnosis (Robbins, Vaghi & Banca, 2019; Strauss et al., 2020) and for research that pursues a dimensional approach to psychopathology (Mcfall & Wollersheim, 1979; Valerius et al., 2008; Jacoby & Abramowitz, 2017).

Ultimately, this is not the last word on the explanations of win-switching or lose-staying, and future work will finesse our accounts. For now, we demonstrate that choice behaviors and latent belief-updating processes can distinguish psychiatric-like symptom phenomenology, even when those phenomena are highly correlated. We believe that we have begun to address this challenge with computational psychiatry — an objective behavioral task (PRL) and fine-grained computational models of behavior (HGF and BGGM). This approach holds promise over the subjective phenomenological approach because of its increased objective precision, and its cross-species translational potential (Corlett & Fletcher, 2021).

## Additional File

The additional file for this article can be found as follows:

10.5334/cpsy.136.s1 Supplemental Materials. Figures S1 to S9 and Tables S1 to S12.
