# Supplementary material for: Excessive Switching in OCD and Paranoia Arises From Different Deficits in Belief-Updating
Source: Comput Psychiatr. 2026 Jun 24;10(1):104–20. doi: 10.5334/cpsy.136 (PMC13308528; doi:10.5334/cpsy.136)
Supplement: Supplemental Materials. — Figures S1 to S9 and Tables S1 to S12. [file cpsy-10-1-136-s1.pdf]

Supplemental Materials: Figures & Tables

Excessive switching in OCD and paranoia arises from different deficits in belief-updating

Charlotte M. Freeland[1], Praveen Suthaharan[1], Santiago Castiello de Obeso[1] , Christopher Pittenger[1,2,3,4,5], Philip R. Corlett[1,2,3]

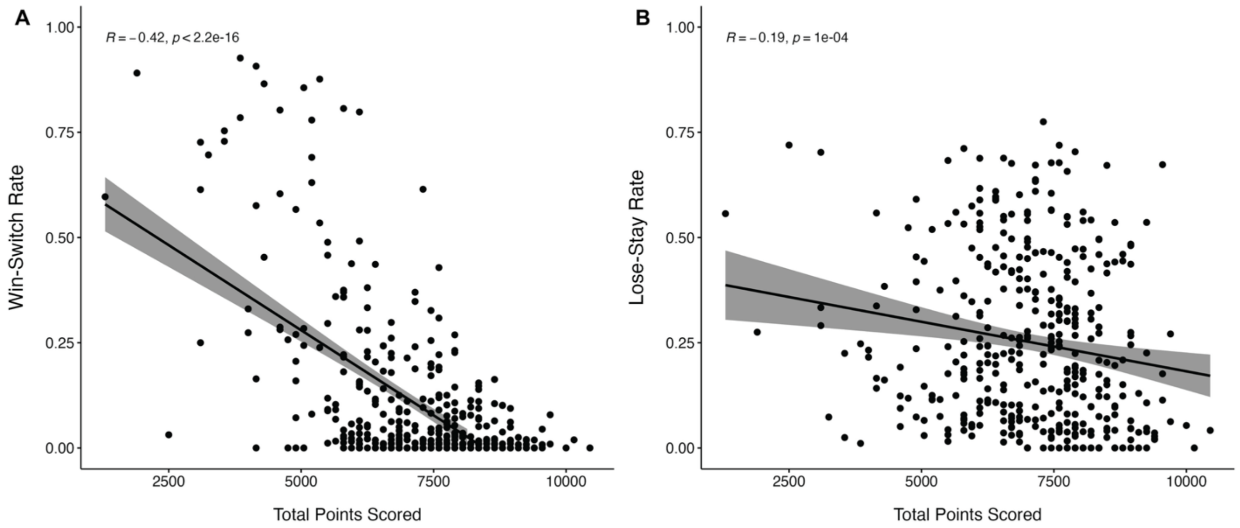

Supplemental Figure 1: Win-switch rate (A) and lose-stay rate (B) were negatively correlated with total points earned in the PRL task (*r* = -0.42, p < 0.001; *r* = -0.19, p < 0.001, respectively), hence reflecting sub-optimal choice strategies.

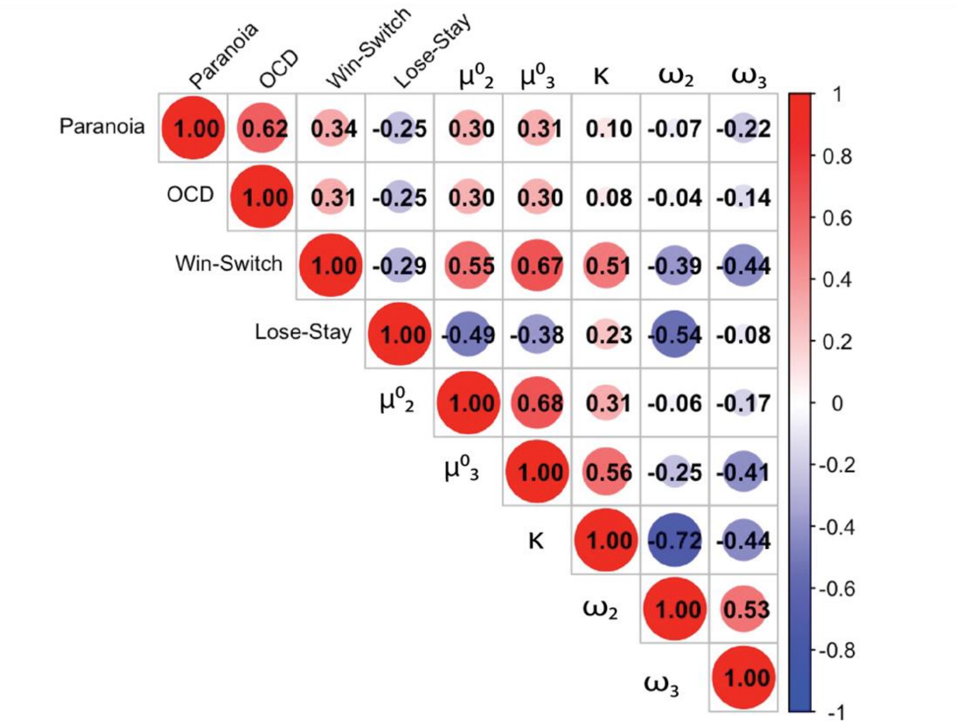

Supplemental Figure 2: Correlation matrix displays positive (red) and negative (blue) Spearman correlations between paranoia and OCD symptoms, win-switch rate, lose-stay rate, and HGF model parameters.

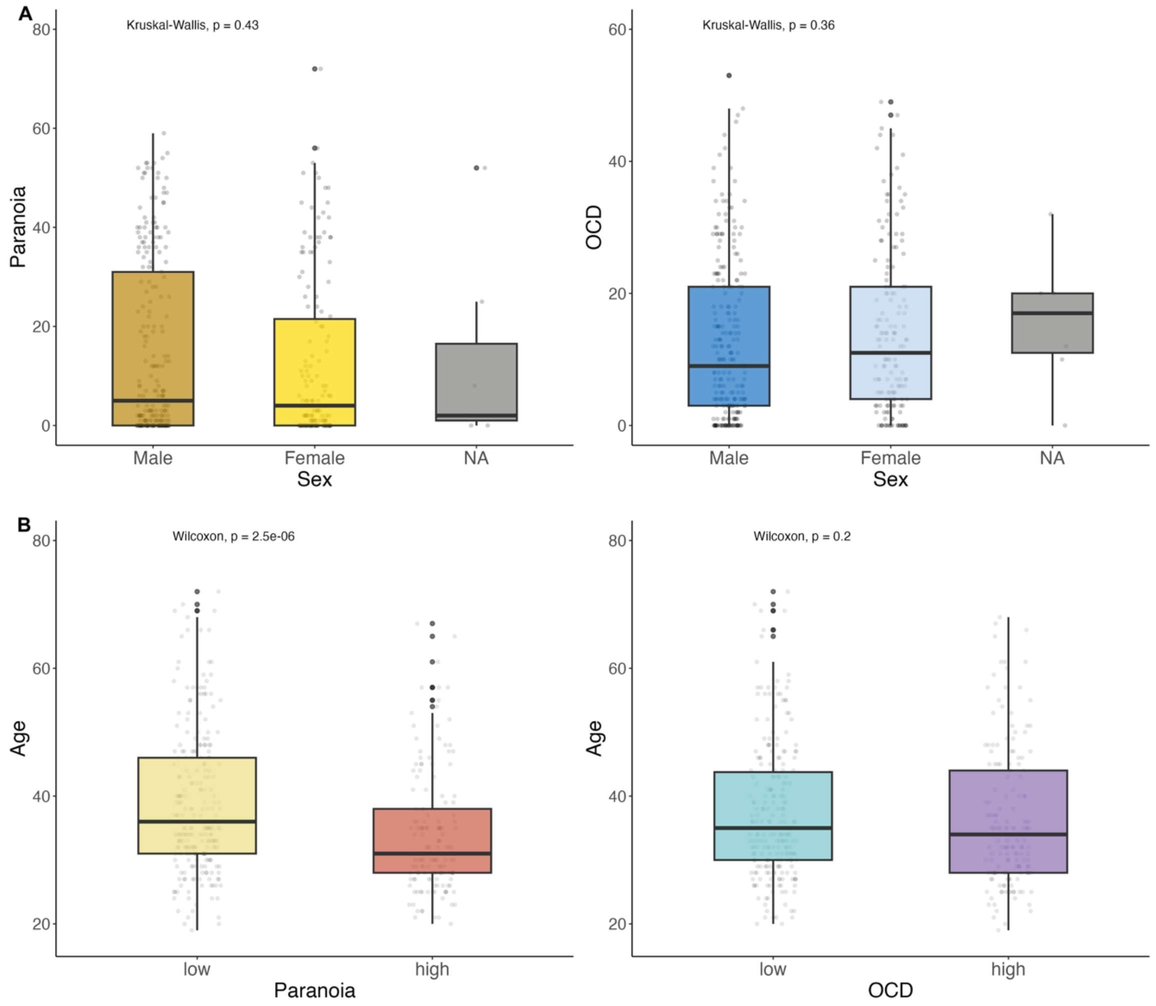

Supplemental Figure 3: (A) There was no effect of participants' sex on paranoia or OCD symptom

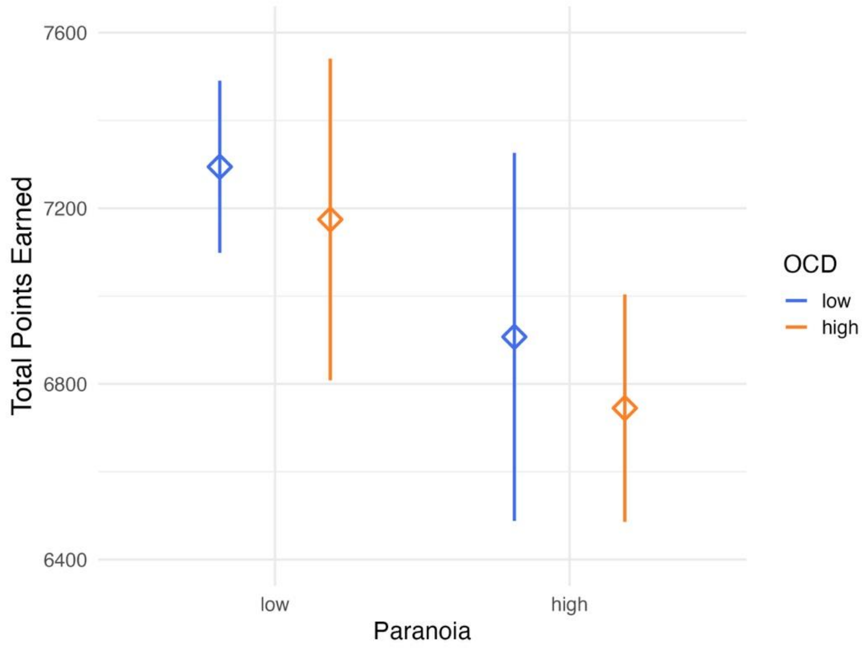

Supplemental Figure 4: There was no significant effect of paranoia group, OCD group, or interaction of paranoia and OCD groups on total points earned on the 3-PRLT. Only individuals with both high paranoia and high OCD earned significantly fewer points than participants with both low paranoia and low OCD ($p = 0.005$).

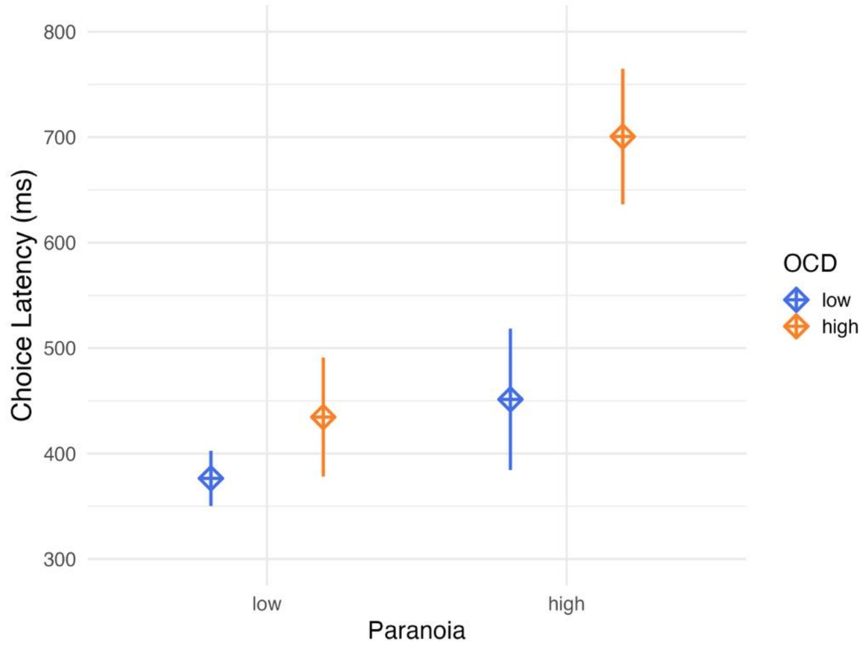

Supplemental Figure 5: Participants with high paranoia took significantly longer to select their choices than participants with low paranoia and regardless of OCD group ($p < 0.001$).

Supplemental Figure 6A: Model diagnostic plots to evaluate assumptions and performance of GLM with a quasibinomial error distribution to model win-switch rate (WSR) as a function of paranoia and OCD groups (see Fig. 3A).

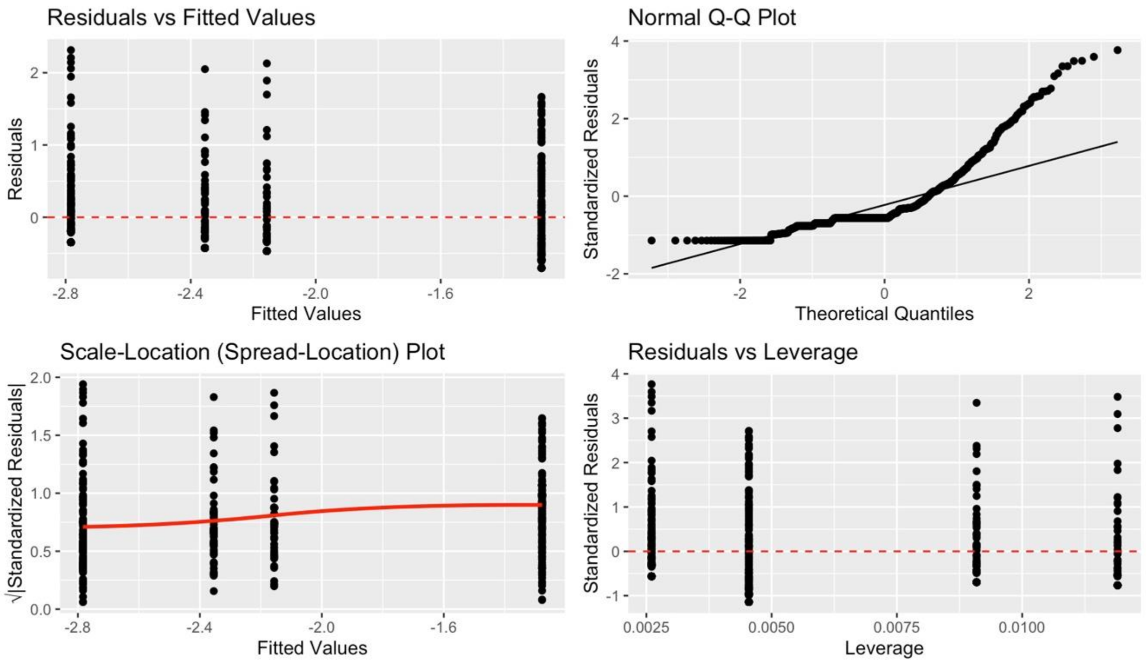

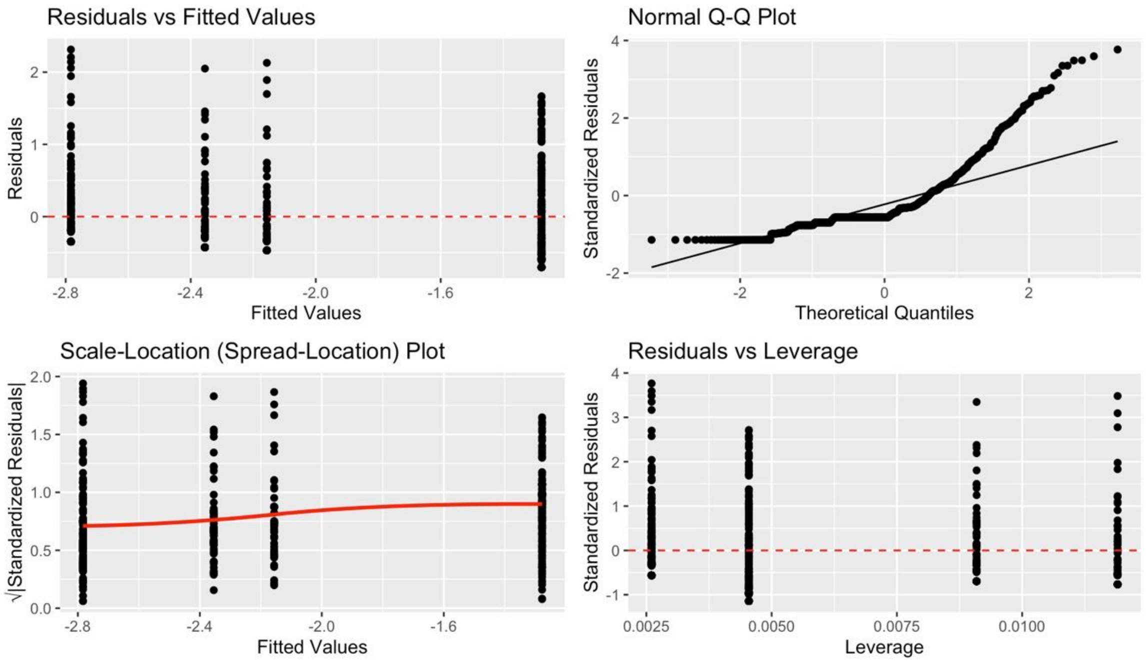

Supplemental Figure 6B: Model diagnostic plots to evaluate assumptions and performance of GLM with a quasibinomial error distribution to model lose-stay rate (LSR) as a function of paranoia and OCD groups (see Fig. 3B).

Supplemental Figure 7A: Model diagnostic plots to evaluate assumptions and performance of GLM with a gaussian error distribution to model $\mu^0{}_2$ as a function of paranoia and OCD groups.

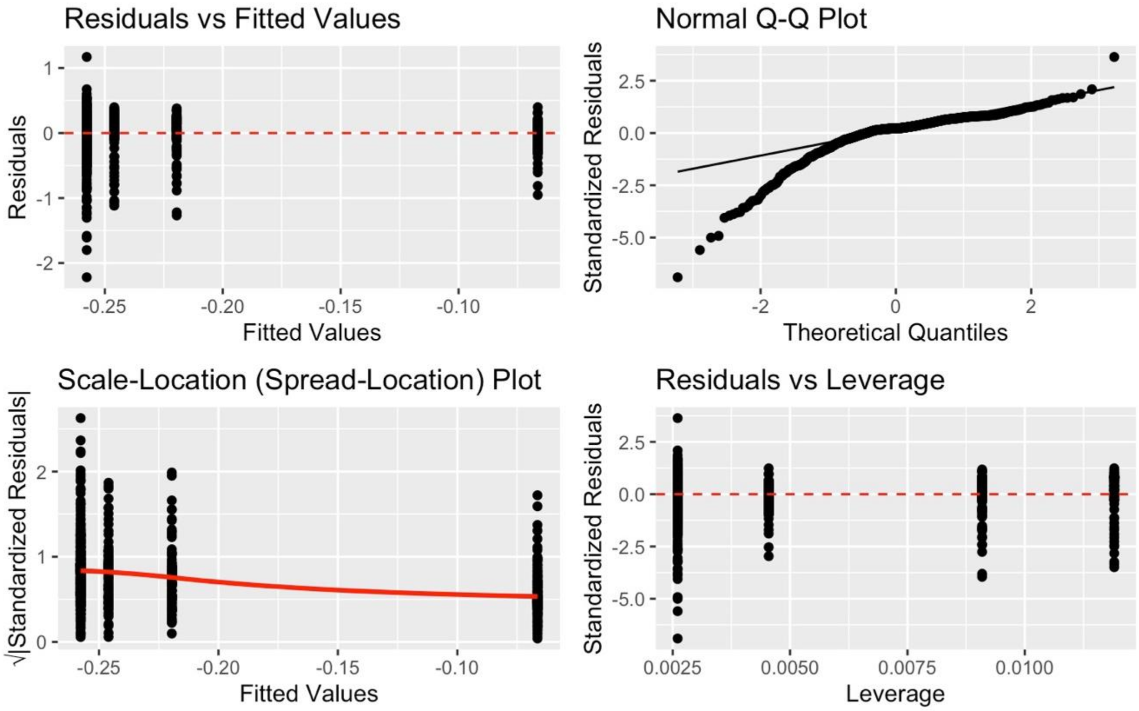

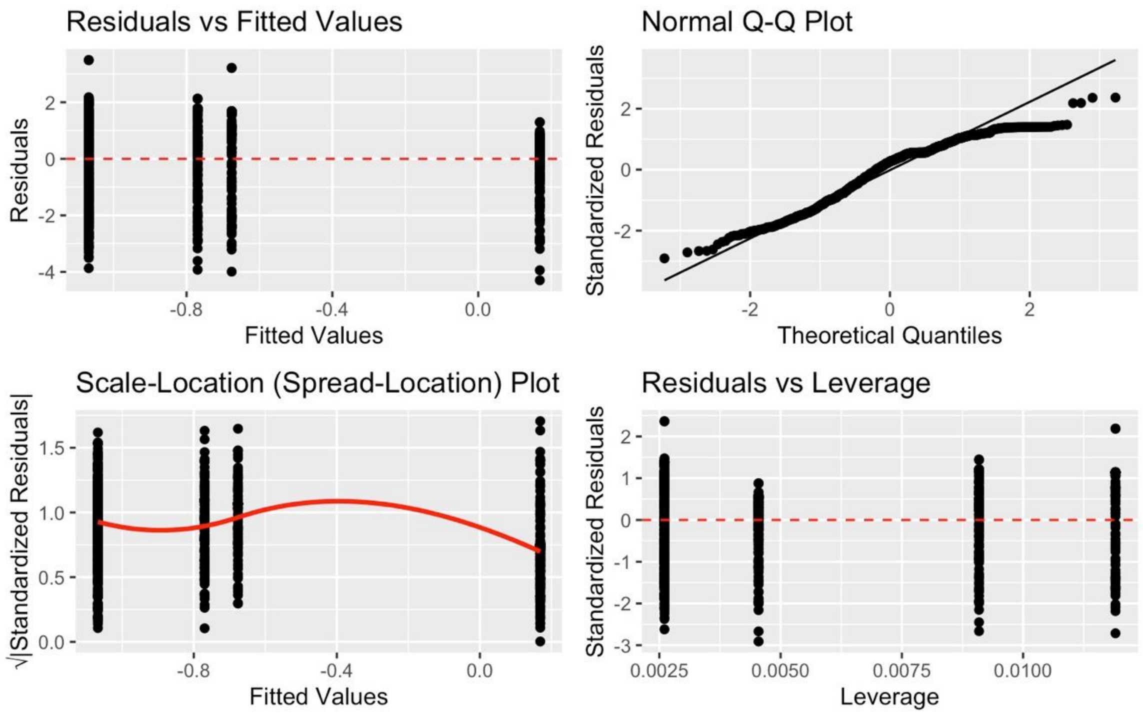

Supplemental Figure 7B: Model diagnostic plots to evaluate assumptions and performance of GLM with a gaussian error distribution to model μ^{0}_{3} as a function of paranoia and OCD groups.

Supplemental Figure 7C: Model diagnostic plots to evaluate assumptions and performance of GLM with a quasibinomial error distribution to model κ as a function of paranoia and OCD groups.

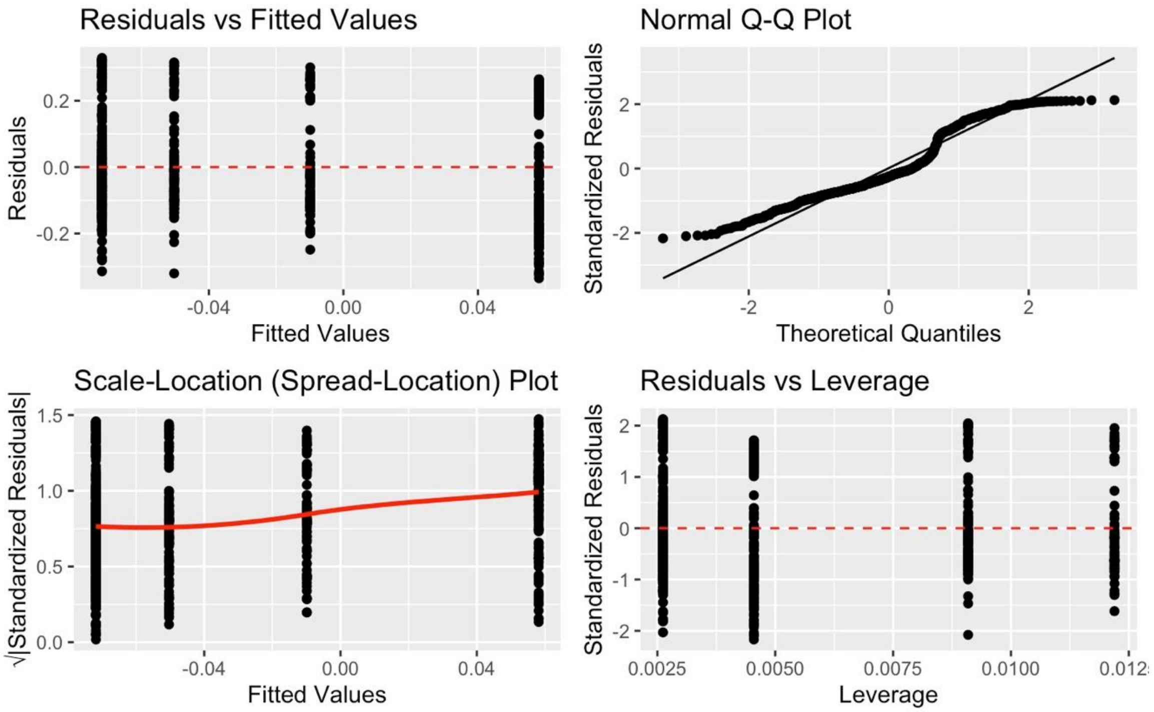

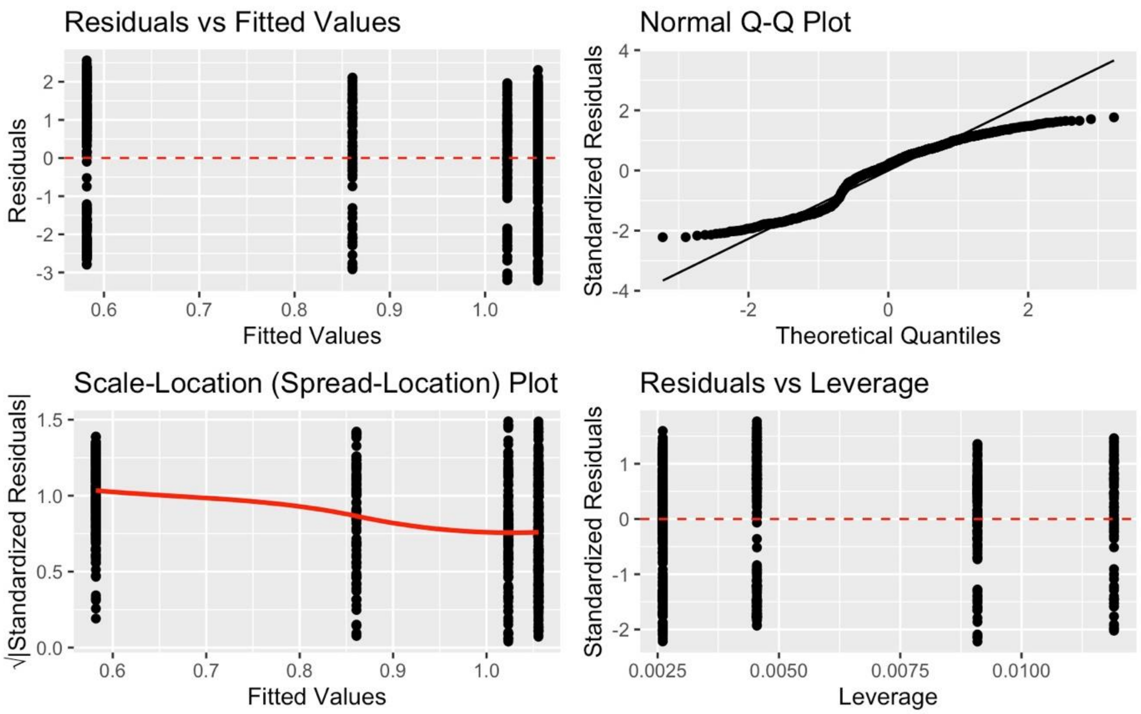

Supplemental Figure 7D: Model diagnostic plots to evaluate assumptions and performance of GLM with a gaussian error distribution to model ω_2 as a function of paranoia and OCD groups.

Supplemental Figure 7E: Model diagnostic plots to evaluate assumptions and performance of GLM with a gaussian error distribution to model ω_3 as a function of paranoia and OCD groups (see Fig. 5).

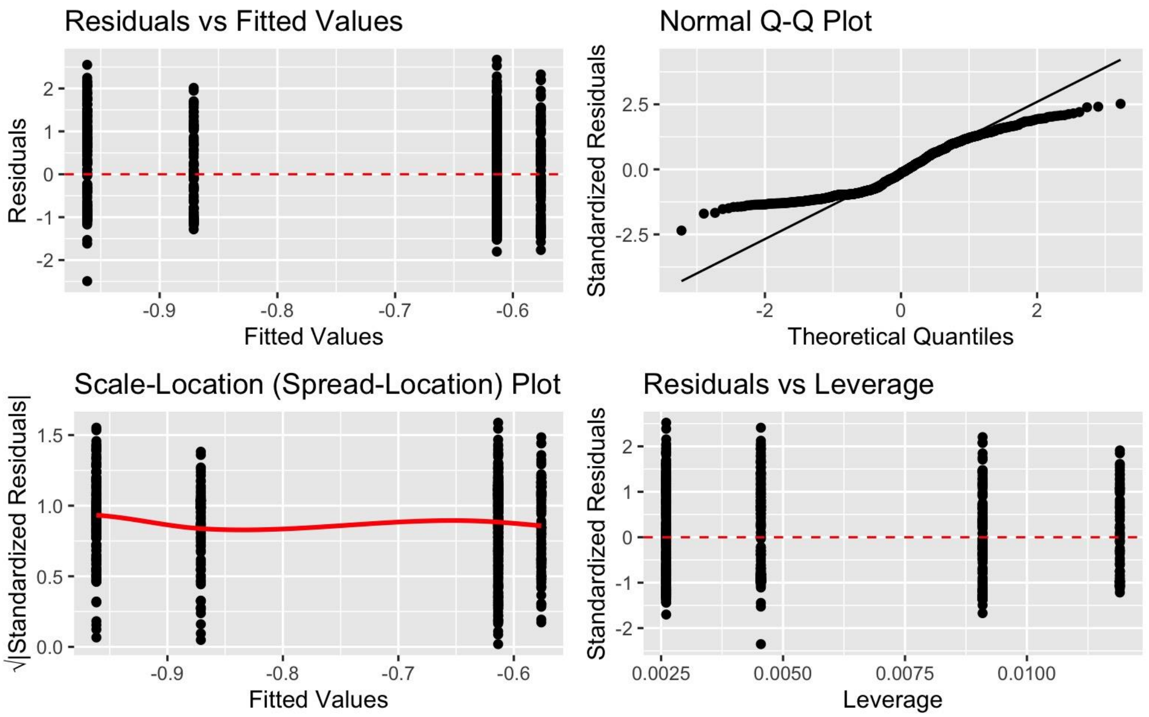

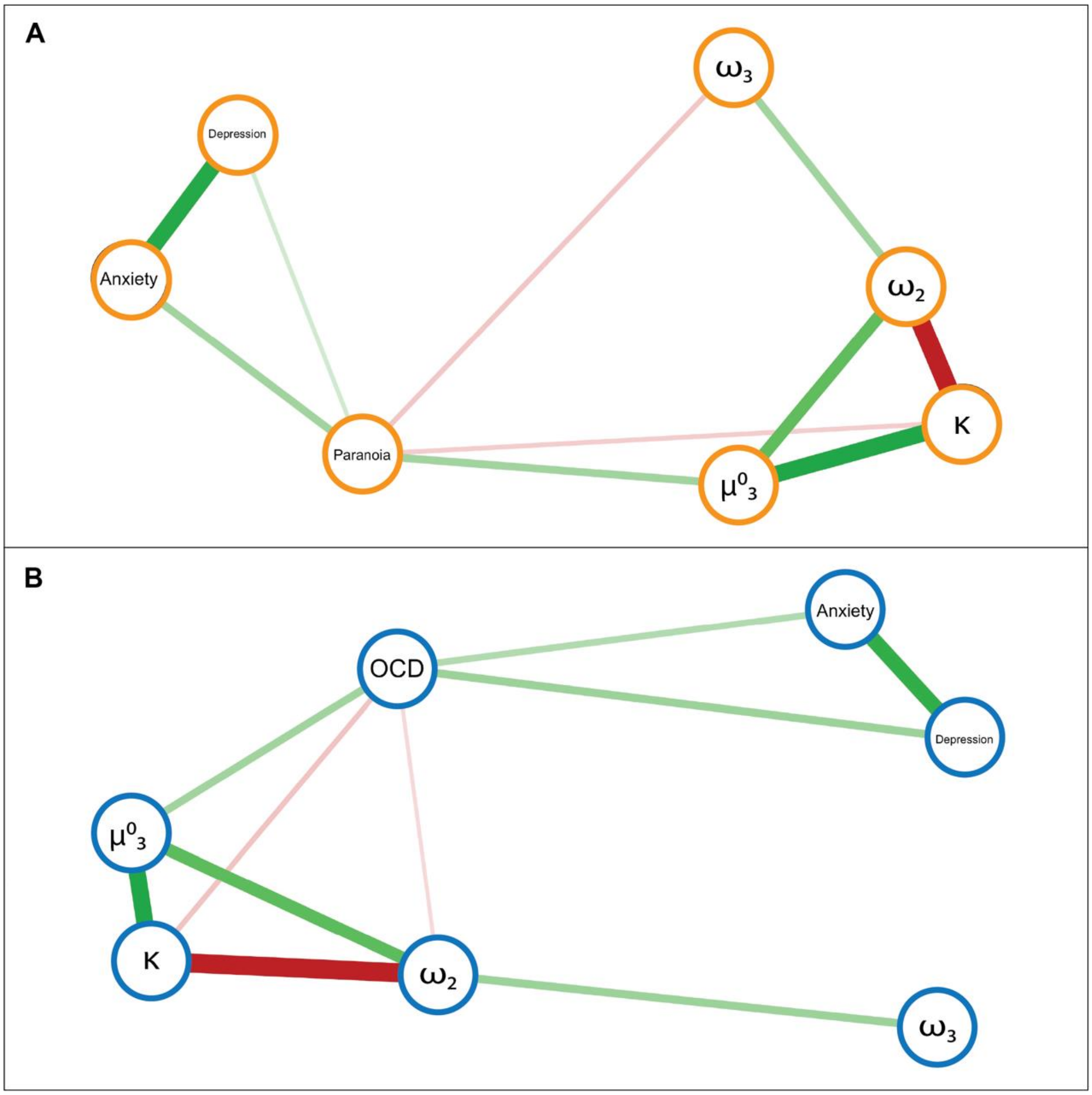

Supplemental Figure 8: While anxiety and depression exhibited strong positive collinearity with paranoia and OCD symptoms, Bayesian Gaussian graphical modeling (BGGM) reveals no significant interaction effects between anxiety and depression in modulating the correlations between HGF model parameters and (A) paranoia or (B) OCD.

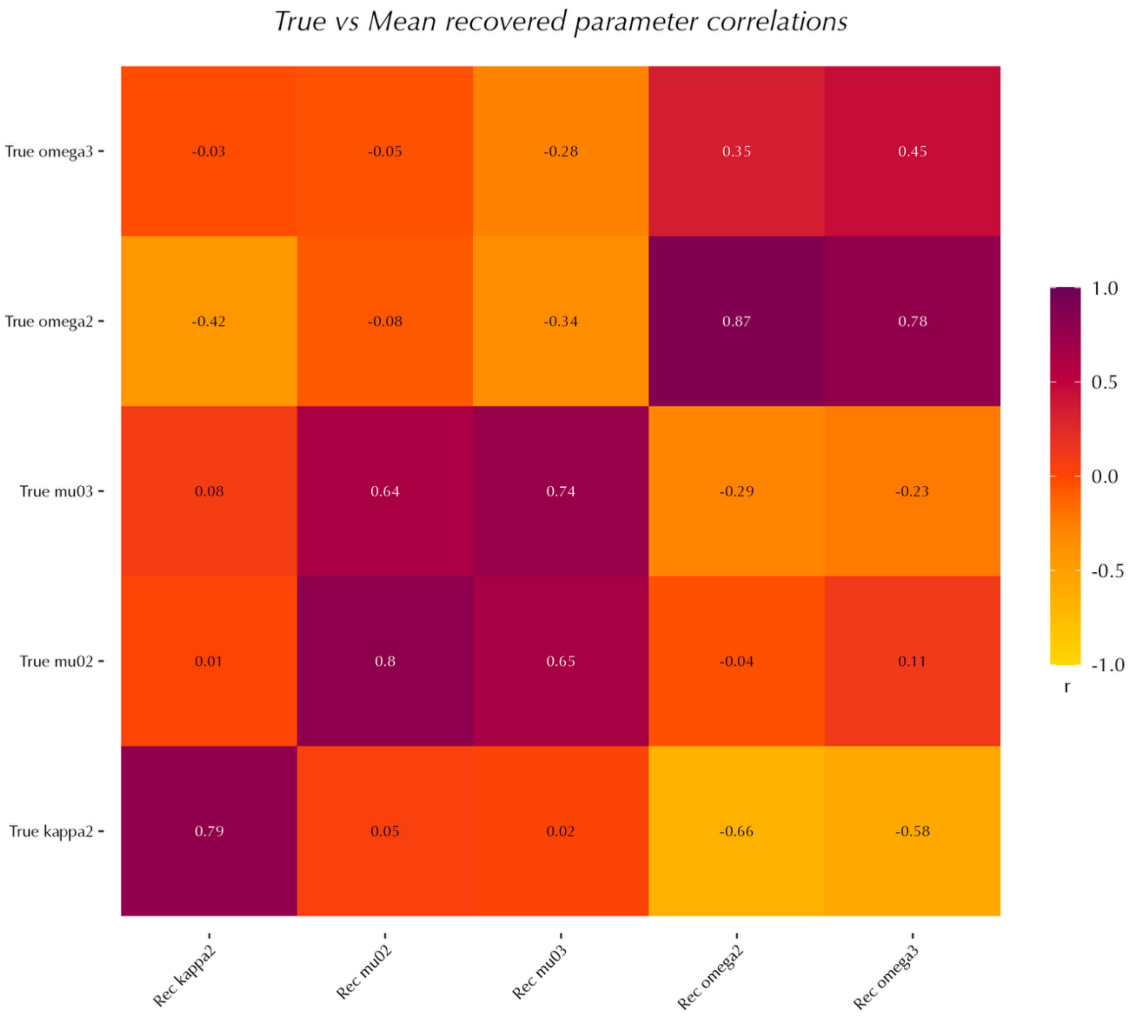

Supplemental Figure 9: Confusion matrix showing correlations between fitted and recovered parameters. Parameter recovery was quantified by correlating mean recovered parameters (across 10 independent simulations) to the original fitted parameters across participants. Mu02 ($\mu^0{}_2$), Mu03 ($\mu^0{}_3$), Kappa2 (κ), Omega2 (ω_2) had robust parameter recovery values (*r's* $\geq$ 0.74). Omega3 (ω_3) was less reliably recovered (*r* = 0.45). These results support the identifiability of the model parameters under this 3-level HGF configuration.

Table S1a. *Generalized linear model predicting win-switch rate in the 3-choice PRL task by paranoia and OCD group (WSR ~ Paranoia group * OCD group).*

Predictor	β	SE	t-ratio[1]	p-value
(Intercept)	-2.78	0.13	-20.77	0.000
Paranoia	0.63	0.26	2.43	**0.015**
OCD	0.43	0.25	1.73	0.084
Paranoia * OCD	0.45	0.35	1.30	0.194

[1] *df* = 794

Table S1b. *Estimated marginal means of win-switch rate for participants assigned to low/high paranoia and low/high OCD groups (see Fig 3A).*

Paranoia	-	OCD	EMM	SE	*df*	Lower CI[2]	Upper CI[2]
low	-	low	0.06	0.13	Inf	0.05	0.08
high	-	low	0.12	0.22	Inf	0.08	0.18
low	-	high	0.10	0.21	Inf	0.06	0.14
high	-	high	0.28	0.10	Inf	0.23	0.34

Note: Results are given on the response scale; EMM: estimated marginal means; SE: standard error of the mean

[2] 95% two-sided confidence interval

Table S1c. *Pairwise contrasts of estimated marginal means for win-switch rate*

Contrasts					Mean difference	SE	z-ratio[3]	p-value[4]
$_P$low	$_O$low	-	$_P$high	$_O$low	0.53	0.26	-2.43	0.071
$_P$low	$_O$low	-	$_P$low	$_O$high	0.65	0.25	-1.73	0.307
$_P$low	$_O$low	-	$_P$high	$_O$high	0.22	0.17	-8.99	**<0.001**
$_P$high	$_O$low	-	$_P$low	$_O$high	1.22	0.30	0.65	0.914
$_P$high	$_O$low	-	$_P$high	$_O$high	0.41	0.24	-3.63	**0.002**
$_P$low	$_O$high	-	$_P$high	$_O$high	0.34	0.23	-4.65	**<0.001**

Notation: $_p$low $_o$low represents subjects with both low paranoia and low OCD. Contrast 1 compares subjects with low paranoia and low OCD to subjects with high paranoia and low OCD.

[3] *df* = Inf

[4] p-value adjusted for multiple comparisons with Tukey method

Table S2a. *Generalized linear model predicting lose-stay rate in the 3-choice PRL task by paranoia and OCD group (LSR ~ Paranoia group * OCD group).*

Predictor	β	SE	t-ratio[1]	p-value
(Intercept)	-0.87	0.05	-16.34	0.000
Paranoia	-0.04	0.13	-0.33	0.743
OCD	-0.15	0.12	-1.26	0.207
Paranoia * OCD	-0.65	0.19	-3.48	**0.001**

[1] *df* = 794

Table S2b. *Estimated marginal means of win-switch rate for participants assigned to low/high paranoia and low/high OCD groups (see Fig. 3B).*

Paranoia - OCD	EMM	SE	*df*	Lower CI [2]	Upper CI [2]
low - low	0.42	0.05	Inf	0.38	0.46
high - low	0.40	0.12	Inf	0.32	0.50
low - high	0.36	0.10	Inf	0.30	0.44
high - high	0.18	0.09	Inf	0.15	0.22

Note: Results are given on the response scale; EMM: estimated marginal mean; SE: standard error of the mean
[2] 95% two-sided confidence interval

Table S2c. *Pairwise contrasts of estimated marginal means for lose-stay rate*

Contrasts	Mean difference	SE	z-ratio[3]	p-value[4]
$_{P}$low $_{O}$low - $_{P}$high $_{O}$low	1.04	0.13	0.33	0.988
$_{P}$low $_{O}$low - $_{P}$low $_{O}$high	1.16	0.12	1.26	0.587
$_{P}$low $_{O}$low - $_{P}$high $_{O}$high	2.32	0.10	8.03	**<0.001**
$_{P}$high $_{O}$low - $_{P}$low $_{O}$high	1.11	0.15	0.68	0.905
$_{P}$high $_{O}$low - $_{P}$high $_{O}$high	2.20	0.15	5.45	**<0.001**
$_{P}$low $_{O}$high - $_{P}$high $_{O}$high	1.99	0.14	5.05	**<0.001**

Notation: $_{p}$low $_{o}$low represents subjects with both low paranoia and low OCD. Contrast 1 compares subjects with low paranoia and low OCD to subjects with high paranoia and low OCD.
[3] *df* = Inf
[4] p-value adjusted for multiple comparisons with Tukey method

Table S3a. *Generalized linear model predicting total points earned in the 3-choice PRL task by paranoia and OCD group (Total points earned ~ Paranoia group * OCD group).*

Model coefficients:	β	SE	t-value	p-value
(Intercept)	7294.53	99.69	73.171	0.000
Paranoia group	-387.39	235.31	-1.646	0.101
OCD group	-119.99	211.27	-0.568	0.570
Paranoia * OCD	-42.16	327.74	-0.129	0.898

Table S3b. *Pairwise contrasts of estimated marginal means for total points earned as a function of paranoia and OCD groups (see Supplemental Fig. 3)*

Contrasts	Mean difference[1]	SE[2]	z-ratio[3]	p-value[4]
$_{P}$low $_{O}$low - $_{P}$high $_{O}$low	387.39	235.31	1.65	0.354
$_{P}$low $_{O}$low - $_{P}$low $_{O}$high	119.99	211.26	0.57	0.942
$_{P}$low $_{O}$low - $_{P}$high $_{O}$high	549.53	165.18	3.33	**0.005**
$_{P}$high $_{O}$low - $_{P}$low $_{O}$high	-267.40	283.07	-0.94	0.781
$_{P}$high $_{O}$low - $_{P}$high $_{O}$high	162.14	250.56	0.65	0.917
$_{P}$low $_{O}$high - $_{P}$high $_{O}$high	429.55	228.13	1.88	0.237

Notation: $_{p}$low $_{o}$low represents subjects with both low paranoia and low OCD. Contrast 1 compares subjects with low paranoia and low OCD to subjects with high paranoia and low OCD.

[1] Estimated marginal mean difference

[2] Standard error

[3] *df* = 794

[4] p-value adjusted for multiple comparisons with Tukey method

Table S4a. *Generalized linear model predicting average choice latency in the 3-choice PRL task by paranoia and OCD groups (Choice latency ~ Paranoia group * OCD group).*

Model coefficients:	β	SE	t-value	p-value
(Intercept)	376.50	13.32	28.274	<0.001
Paranoia group	74.90	36.64	2.044	**0.041**
OCD group	58.11	31.66	1.836	0.067
Paranoia * OCD	191.04	56.91	3.357	**<0.001**

Table S4b. *Pairwise contrasts of estimated marginal means for choice latency as a function of low/high paranoia and OCD groups (see Supplemental Fig. 4)*

Contrasts	Mean difference[1]	SE[1]	z-ratio[2]	p-value[3]
$_{P}$low $_{O}$low - $_{P}$high $_{O}$low	-74.90	36.64	-2.04	0.173
$_{P}$low $_{O}$low - $_{P}$low $_{O}$high	-58.11	31.66	-1.84	0.257
$_{P}$low $_{O}$low - $_{P}$high $_{O}$high	-324.05	35.34	-9.17	**<0.001**
$_{P}$high $_{O}$low - $_{P}$low $_{O}$high	16.79	44.61	0.38	0.982
$_{P}$high $_{O}$low - $_{P}$high $_{O}$high	-249.15	47.29	-5.27	**<0.001**
$_{P}$low $_{O}$high - $_{P}$high $_{O}$high	-265.94	43.55	-6.11	**<0.001**

Notation: $_{p}$low $_{o}$low represents subjects with both low paranoia and low OCD. Contrast 1 compares subjects with low paranoia and low OCD to subjects with high paranoia and low OCD.

[1] Estimated marginal mean difference and standard error (SE) values for choice latency are reported in milliseconds

[2] *df* = 794

[3] p-value adjusted for multiple comparisons with Tukey method

Table S5. *BGGM summary table of relationships between paranoia, OCD, win-switch rate (WSR) and lose-stay rate (LSR) (see Fig. 4). For all posterior probability hypothesis comparisons, see “Figure-4-bggm-summary.txt”.*

Relation	*P*(H0)	*P*(H1)	*P*(H2)
Paranoia -- OCD	0.000	**1.000**	0.000
Paranoia -- WSR	0.000	**1.000**	0.000
OCD -- WSR	0.186	**0.806**	0.008
Paranoia -- LSR	0.283	0.010	**0.707**
OCD -- LSR	0.440	0.011	**0.549**
WSR -- LSR	0.195	0.008	**0.797**

Table S6a. *Generalized linear model predicting HGF model parameter* μ^0_2 *in the 3-choice PRL task as a function of paranoia group and OCD group.*

Predictor	β	SE	t-ratio[1]	p-value
(Intercept)	-0.26	0.02	-15.69	0.000
Paranoia	0.01	0.04	0.30	0.764
OCD	0.04	0.04	1.10	0.273
Paranoia * OCD	0.14	0.05	2.62	**0.009**

[1] *df* = 794

Table S6b. *Estimated marginal means of parameter* μ^0_2 *for participants assigned to low/high paranoia and low/high OCD groups (see Fig. 5, bottom left).*

Paranoia	-	OCD	EMM	SE	*df*	Lower CI [2]	Upper CI [2]
low	-	low	-0.26	0.02	794	-0.29	-0.23
high	-	low	-0.25	0.04	794	-0.31	-0.18
low	-	high	-0.22	0.03	794	-0.28	-0.16
high	-	high	-0.07	0.02	794	-0.11	-0.02

Note: Results are given on the response scale; EMM: estimated marginal means; SE: standard error of the mean

[2] 95% two-sided confidence interval

Table S6c. *Pairwise contrasts of estimated marginal means for parameter* μ^0_2

Contrasts					Mean difference	SE	t-ratio[3]	p-value[4]
$_P$low	$_O$low	-	$_P$high	$_O$low	-0.01	0.04	-0.30	0.991
$_P$low	$_O$low	-	$_P$low	$_O$high	-0.04	0.03	-1.10	0.692
$_P$low	$_O$low	-	$_P$high	$_O$high	-0.19	0.03	-7.03	**<0.001**
$_P$high	$_O$low	-	$_P$low	$_O$high	-0.03	0.05	-0.57	0.942
$_P$high	$_O$low	-	$_P$high	$_O$high	-0.18	0.04	-4.35	**<0.001**
$_P$low	$_O$high	-	$_P$high	$_O$high	-0.15	0.04	-4.07	**<0.001**

Notation: $_p$low $_o$low represents subjects with both low paranoia and low OCD. Contrast 1 compares subjects with low paranoia and low OCD to subjects with high paranoia and low OCD.

[3] *df* = 794

[4] p-value adjusted for multiple comparisons with Tukey method

Table S7a. *Generalized linear model predicting HGF model parameter ω_2 in the 3-choice PRL task as a function of paranoia group and OCD group.*

Predictor	β	SE	t-ratio[1]	p-value
(Intercept)	1.06	0.07	14.25	0.000
Paranoia	-0.20	0.18	-1.11	0.266
OCD	-0.03	0.16	-0.21	0.837
Paranoia * OCD	-0.25	0.24	-1.01	0.312

[1] *df* = 794

Table S7b. *Estimated marginal means of parameter ω_2 for participants assigned to low/high paranoia and low/high OCD groups (see Fig. 5, bottom right).*

Paranoia	-	OCD	EMM	SE	*df*	Lower CI[2]	Upper CI[2]
low	-	low	1.06	0.07	794	0.91	1.20
high	-	low	0.86	0.16	794	0.55	1.17
low	-	high	1.02	0.14	794	0.75	1.30
high	-	high	0.58	0.10	794	0.39	0.77

Note: Results are given on the response scale; EMM: estimated marginal means; SE: standard error of the mean

[2] 95% two-sided confidence interval

Table S7c. *Pairwise contrasts of estimated marginal means for parameter ω_2*

Contrasts					Mean difference	SE	t-ratio[3]	p-value[4]
$_P$low	$_O$low	-	$_P$high	$_O$low	0.19	0.17	1.11	0.681
$_P$low	$_O$low	-	$_P$low	$_O$high	0.03	0.16	0.21	0.997
$_P$low	$_O$low	-	$_P$high	$_O$high	0.47	0.12	3.86	**0.001**
$_P$high	$_O$low	-	$_P$low	$_O$high	-0.16	0.21	-0.77	0.867
$_P$high	$_O$low	-	$_P$high	$_O$high	0.28	0.19	1.50	0.440
$_P$low	$_O$high	-	$_P$high	$_O$high	0.44	0.17	2.60	**0.046**

Notation: $_p$low $_o$low represents subjects with both low paranoia and low OCD. Contrast 1 compares subjects with low paranoia and low OCD to subjects with high paranoia and low OCD.

[3] *df* = 794

[4] p-value adjusted for multiple comparisons with Tukey method

Table S8a. *Generalized linear model predicting HGF model parameter* **κ** *in the 3-choice PRL task as a function of paranoia group and OCD group.*

Predictor	β	SE	t-ratio[1]	p-value
(Intercept)	-0.07	0.02	-4.53	0.000
Paranoia	0.06	0.04	1.64	0.101
OCD	0.02	0.03	0.64	0.522
Paranoia * OCD	0.05	0.05	0.89	0.372

[1]*df* = Inf

Table S8b. *Estimated marginal means of parameter* **κ** for participants assigned to low/high paranoia and low/high OCD groups *(see Fig. 5, middle).*

Paranoia	-	OCD	EMM	SE	*df*	Lower CI [2]	Upper CI [2]
low	-	low	0.93	0.02	Inf	0.91	0.96
high	-	low	0.99	0.03	Inf	0.92	1.06
low	-	high	0.95	0.03	Inf	0.90	1.01
high	-	high	1.06	0.02	Inf	1.02	1.11

Note: Results are given on the response scale; EMM: estimated marginal means; SE: standard error of the mean

[2] 95% two-sided confidence interval

Table S8c. *Pairwise contrasts of estimated marginal means for parameter* **κ**

Contrasts				Mean difference	SE	z-ratio[3]	p-value[4]
$_{P}$low	$_{O}$low	-	$_{P}$high	0.94	0.04	-1.64	0.354
$_{P}$low	$_{O}$low	-	$_{P}$low	0.98	0.03	0.64	0.919
$_{P}$low	$_{O}$low	-	$_{P}$high	0.88	0.03	-4.96	**<0.001**
$_{P}$high	$_{O}$low	-	$_{P}$low	1.04	0.05	0.90	0.807
$_{P}$high	$_{O}$low	-	$_{P}$high	0.93	0.04	-1.70	0.324
$_{P}$low	$_{O}$high	-	$_{P}$high	0.90	0.04	-3.00	**0.014**

Notation: $_{p}$low $_{o}$low represents subjects with both low paranoia and low OCD. Contrast 1 compares subjects with low paranoia and low OCD to subjects with high paranoia and low OCD.

[3] *df* = Inf

[4] p-value adjusted for multiple comparisons with Tukey method

Table S9a. *Generalized linear model predicting HGF model parameter μ^0_3 in the 3-choice PRL task as a function of paranoia group and OCD group.*

Predictor	β	SE	t-ratio[1]	p-value
(Intercept)	-1.07	0.08	-14.14	0.000
Paranoia	0.40	0.18	2.20	**0.028**
OCD	0.30	0.16	1.87	0.063
Paranoia * OCD	0.55	0.25	2.20	**0.028**

[1]*df* = 794

Table S9b. *Estimated marginal means of parameter μ^0_3* for participants assigned to low/high paranoia and low/high OCD groups *(see Fig. 5, top left).*

Paranoia	-	OCD	EMM	SE	*df*	Lower CI [2]	Upper CI [2]
low	-	low	-1.07	0.08	794	-1.22	-0.92
high	-	low	-0.68	0.16	794	-0.99	-0.36
low	-	high	-0.77	0.14	794	-1.05	-0.49
high	-	high	0.17	0.10	794	-0.03	0.36

Note: Results are given on the response scale. EMM: estimated marginal means; SE: standard error of the mean

[2] 95% two-sided confidence interval

Table S9c. *Pairwise contrasts of estimated marginal means for parameter μ^0_3*

Contrasts					Mean difference	SE	t-ratio[3]	p-value[4]
$_P$low	$_O$low	-	$_P$high	$_O$low	-0.39	0.18	-2.20	0.125
$_P$low	$_O$low	-	$_P$low	$_O$high	-0.30	0.16	-1.87	0.244
$_P$low	$_O$low	-	$_P$high	$_O$high	-1.24	0.13	-9.88	**<0.001**
$_P$high	$_O$low	-	$_P$low	$_O$high	0.09	0.21	0.44	0.972
$_P$high	$_O$low	-	$_P$high	$_O$high	-0.85	0.19	-4.45	**<0.001**
$_P$low	$_O$high	-	$_P$high	$_O$high	-0.94	0.17	-5.43	**<0.001**

Notation: $_p$low $_o$low represents subjects with both low paranoia and low OCD. Contrast 1 compares subjects with both low paranoia and low OCD to subjects with both high paranoia and low OCD.

[3] *df* = 794

[4] p-value adjusted for multiple comparisons with Tukey method

Table S10a. *Generalized linear model predicting HGF model parameter for ω_3 in the 3-choice PRL task as a function of paranoia group and OCD group.*

Predictor	β	SE	t-ratio[1]	p-value
(Intercept)	-0.61	0.05	-11.34	0.000
Paranoia	-0.26	0.13	-2.02	**0.044**
OCD	0.04	0.11	0.33	0.745
Paranoia * OCD	-0.13	0.18	-0.72	0.473

[1] *df* = 794

Table S10b. *Estimated marginal means of parameter ω_3 by low/high paranoia and low/high OCD groups (see Fig. 5, top right).*

Paranoia	-	OCD	EMM	SE	DF	Lower CI [2]	Upper CI [2]
low	-	low	-0.61	0.05	794	-0.72	-0.51
high	-	low	-0.87	0.12	794	-1.10	-0.64
low	-	high	-0.58	0.10	794	-0.77	-0.38
high	-	high	-0.96	0.07	794	-1.10	-0.82

Note: Results are given on the response scale; EMM: estimated marginal means; SE: standard error of the mean

[2] 95% two-sided confidence interval

Table S10c. *Pairwise contrasts of estimated marginal means for parameter ω_3*

Contrasts					Mean difference	SE	t-ratio[3]	p-value[4]
$_P$low	$_O$low	-	$_P$high	$_O$low	0.26	0.13	2.02	0.183
$_P$low	$_O$low	-	$_P$low	$_O$high	-0.04	0.11	-0.33	0.988
$_P$low	$_O$low	-	$_P$high	$_O$high	0.35	0.09	3.88	**0.001**
$_P$high	$_O$low	-	$_P$low	$_O$high	-0.29	0.15	-1.92	0.221
$_P$high	$_O$low	-	$_P$high	$_O$high	0.09	0.14	0.66	0.910
$_P$low	$_O$high	-	$_P$high	$_O$high	0.39	0.12	3.11	**0.010**

Notation: $_P$low $_O$low represents subjects with both low paranoia and low OCD. Contrast 1 compares subjects with low paranoia and low OCD to subjects with high paranoia and low OCD.

[3] *df* = 794

[4] p-value adjusted for multiple comparisons with Tukey method

Table S11. *BGGM summary table of relationships between paranoia, OCD, and HGF model parameters (see Fig. 6). Posterior hypothesis probabilities for a null relation (P*(H0)), a positive relation *(P*(H1)), or a negative relation *(P*(H2)) to the alternative hypothesis. *For all posterior probability hypothesis comparisons, see "Figure-6-bggm-summary.txt".*

	Paranoia	OCD
μ^0_2	**P(H0) = 0.852**<br>P(H1) = 0.139<br>P(H2) = 0.009	**P(H0) = 0.985**<br>P(H1) = 0.009<br>P(H2) = 0.006
μ^0_3	P(H0) = 0.419<br>P(H1) = 0.572<br>P(H2) = 0.009	P(H0) = 0.010<br>**P(H1) = 0.988**<br>P(H2) = 0.001
κ	**P(H0) = 0.837**<br>P(H1) = 0.009<br>P(H2) = 0.155	P(H0) = 0.042<br>P(H1) = 0.003<br>**P(H2) = 0.955**
ω_2	**P(H0) = 0.964**<br>P(H1) = 0.006<br>P(H2) = 0.030	P(H0) = 0.163<br>P(H1) = 0.007<br>**P(H2) = 0.830**
ω_3	P(H0) = 0.001<br>P(H1) = 0.000<br>**P(H2) = 0.999**	**P(H0) = 0.978**<br>P(H1) = 0.016<br>P(H2) = 0.006

Table S12a. *BGGM summary table of relationships between paranoia, anxiety, depression, and HGF model parameters (see Supplementary Fig. 8A).*

	Paranoia	Anxiety	Depression
Anxiety	*P*(H1) = 1.000		*P*(H1) = 1.000
Depression	*P*(H1) = 0.976	*P*(H1) = 1.000	
μ^0_3	*P*(H1) = 1.000	*P*(H0) = 0.972	*P*(H0) = 0.983
κ	*P*(H2) = 0.972	*P*(H0) = 0.985	*P*(H0) = 0.963
ω_2	*P*(H0) = 0.859	*P*(H0) = 0.984	*P*(H0) = 0.849
ω_3	*P*(H2) = 1.000	*P*(H0) = 0.975	*P*(H0) = 0.983

Table S12b. *BGGM summary table of relationships between OCD, anxiety, depression, and HGF model parameters (see Supplementary Fig. 8B).*

	OCD	Anxiety	Depression
Anxiety	*P*(H1) = 1.000		*P*(H1) = 1.000
Depression	*P*(H1) = 1.000	*P*(H1) = 1.000	
μ^0_3	*P*(H1) = 1.000	*P*(H0) = 0.960	*P*(H0) = 0.916
κ	*P*(H2) = 0.999	*P*(H0) = 0.983	*P*(H0) = 0.984
ω_2	*P*(H2) = 0.761	*P*(H0) = 0.983	*P*(H0) = 0.961
ω_3	*P*(H0) = 0.923	*P*(H0) = 0.985	*P*(H0) = 0.984
