# Supplementary material for: Excessive Switching in OCD and Paranoia Arises From Different Deficits in Belief-Updating
Source: Comput Psychiatr. 2026 Jun 24;10(1):104–20. doi: 10.5334/cpsy.136 (PMC13308528; doi:10.5334/cpsy.136)
Supplement: Peer review history

# Reviewer report: Round 1, Reviewer A

## Recommendation:

Accept with Major Revision

## Comments/Explanation (required). Please include references to any minor or major revisions required, as well as addressing any issues with language clarity. Authors will see these comments:

Freeland et al. try to tackle an important problem of differentiating belief updating deficits that relate to OCD and paranoia. To do so they re-analyze previously published data on a 3-choice probabilistic learning task across social and non-social domains. The strength of the paper is the combination of an HGF model with Bayesian network analysis - Bayesian Gaussian Graphical Modeling (BGGM) to try to uncover subtle differences in how updating of individuals with OCD differentiates from those with paranoia.

Behavioural findings show that both paranoia and OCD symptom severities are associated with increased switching both after wins and after losses with a difference between the two symptom domains (however it is not clear from the text or the figure in which direction). Using BGGM the authors show that there might be slight differences in the strength with which each symptom domain relates to switching behavior.

The authors then use the HGF to explore the subtle nuances in trial-by-trial belief updates and find important differences between how the symptom domains relate to the meta-volatility (“omega3”) and volatility (“omega2”) parameters.

While these results tell a compelling story, I am not yet convinced that the model parameters can be taken at face value (are well-defined). This can severely limit the conclusions drawn from this analysis and the subsequent BGGM analysis.

First, parameters such as “inital mu2”, “omega2” and “kappa” all explain very similar belief updating dynamics (as do “initial mu3” and “omega3”) and are (in my experience) very hard to differentiate. That being said, perhaps a 3-choice task with 320 trials is enough to do so. I suggest that the authors show to what degree the model parameters are recoverable (by simulating + refitting).

Second, it is not clear that this is the most parsimonious model that the authors could use for this task. Showing that this model outperforms simpler HGF models with less parameters would be helpful (or at least provide a theoretical argument why such a complex model is necessary to use).

Third, one important parameter that always leads to more overall switching (and more stochastic behaviour overall) is the (inverse) temperature parameter. It is not clear whether the authors included it in the analysis, but if they want to claim that what they find is not related to noise unexplained by the model, I would argue that in needs to be included as a free parameter in the model. The noise parameter should also appear in the BGGM analysis that includes all HGF parameters.

Minor comments:

The authors use the scale for OCD and paranoia in various ways – sometimes they use cutoff points to define two categories of clinical groups (“high paranoia” and “low paranoia”), sometimes four (e.g. Figure 2B) and sometimes use continuous measures (in the network analysis).

This is also mixed up when results are presented adding to the confusion about which variables were used:

e.g.

Line 214: “Consistent with these findings, we observed a 214 greater rate of win-switching in participants with high paranoia, compared to those with low 215 paranoia symptom scores”

line 220: “Furthermore, win-switching significantly increased 220 as both paranoia and OCD symptom scores increased across the clinical continuum”.

It is unclear why this is the case and why for instance continuous measures are not used throughout the MS, since binarizing continuous variables is throwing data away. It would be helpful if the choice of the number of categories is explained and consistent with the language used to describe the results.

When describing the level structure of the HGF model, levels in the figure and text do not match.

# Reviewer report: Round 1, Reviewer D

## Recommendation:

Accept with Major Revision

## Comments/Explanation (required). Please include references to any minor or major revisions required, as well as addressing any issues with language clarity. Authors will see these comments:

The authors present original cognitive modelling work (with the Hierarchical Gaussian Filter (HGF) as their model of choice) of a pre-existing probabilistic reversal learning (PRL) dataset, showing that paranoia and OCD (correlated psychiatric conditions that are hard to disentangle) differ in cognitive parameter estimates on an experimental task. The work is thorough, mostly methodologically explicit and sound, and relies on standard methods in computational psychiatry; it is well motivated and contributes to the understanding of these two prevalent conditions. Additionally, the paper applies a visualisation method (Bayesian Gaussian Graphical Modelling, BGGM) which seems to make clear an otherwise hidden statistical pattern in the data. I therefore find the work valuable and would encourage its publication.

There are, however, quite a few issues that would first need to be addressed before publication, which I describe in the attached document. This particularly relates to the cognitive model and the statistical methods being rather under-described, to a lack of appreciation and clarity regarding limitations of the study, and to a lack of methodological checks and care.

# Reviewer report: Round 2, Reviewer A

## Recommendation:

Accept (No Revisions Necessary)

## Comments/Explanation (required). Please include references to any minor or major revisions required, as well as addressing any issues with language clarity. Authors will see these comments:

Review of Excessive switching in OCD and paranoia arises from different deficits in belief-updating (Round 2)

The authors have carefully incorporated or responded to the points made in my original review, and I am happy to recommend the article for publication. Below are some minor points which the authors may find useful for a final, minor revision:

I appreciate the now expanded description of BGGM, which is very helpful and interesting.

I appreciate that it has been clarified that the HGF parameters were not obtained in this study, but were rather estimates made in a previous experiment. This of course renders many of my previous comments largely irrelevant (such as about parameter recovery and other quality checks of the parameter estimates). I apologize of the confusion, to the degree that it was due to inattentiveness on my part.

In figure five, the omega parameters seem to have a superscript 0, which I expect is a typo.

On line 168, the authors call the HGF a (hierarchical) Bayesian model, and on line 946, a “hierarchical computational approach”. This is accurate, but ambiguous, because the term “hierarchical Bayesian model” also refers to using hierarchical Bayesian methods for fitting models to data model (the BGGM is for example a Bayesian model too, although not hierarchical; a mixed-effects model in brms would also be a hierarchical Bayesian computational model), which can also be done with other types of cognitive models, such as reinforcement learning (see e.g. hBayesDM for examples of this). In order to disambiguate, the authors might call the HGF a “Bayesian mind model”, or, in full, a “variational Bayesian mind predictive processing model of perception and learning”, or something similar.

The authors use an advanced Bayesian network or regressions, but also use a frequentist lme4 method earlier in the article. It makes one wonder why the authors didn’t use Bayesian methods all the way through the article. There is no immediate need to change this, but the authors may consider very briefly commenting on it.

In line 182, the authors claim that Bayesian mind models are “better equipped to capture dynamic trial-by-trial choices and behavioral changes in response to task volatility (e.g., contingency reversal events), and to infer latent processes that lead to distinct behavioral strategies (i.e., win-switching, lose-staying)”. They authors may considering supporting this with one or more references; I am sure there are or could in principle be hierarchical reinforcement learning models appropriate for volatile environments too (although of course the HGF is specifically made for this).

Finally, although the strong correlation between omega3 and kappa is not due to methodological choices that the authors have made (since they use precomputed parameters), they might still comment on the possibility that this strong correlation indicates unidentifiability between the parameters. If this is the case, the correlation found by the BGGM is not empirical, but a consequence of the original fitting procedure. I don’t know if the original authors did parameter recovery studies or similar to test for this, but the authors might report whether it was done, or whether such unidentifiability are reported for similar HGF models and for similar datasets.

Other than these points, I find all my comments appropriately addressed, and congratulate the authors on thorough and clear work.

# Reviewer report: Round 2, Reviewer B

## Recommendation:

Accept with Major Revision

## Comments/Explanation (required). Please include references to any minor or major revisions required, as well as addressing any issues with language clarity. Authors will see these comments:

The authors did an impressive amount of work to improve the readability and comprehension of the manuscript. I find the results of the OCD and paranoia on win-shift behaviour compelling and worth publishing.

However, as I clarify below, my main concerns that relate to the quality of the model used (including an inverse temperature parameter and making sure that the model used is well defined) are not addressed.

The authors write the following in the response to the parameter recovery issue:

“The present study analyzed this publicly available and peer-reviewed dataset, including the previously computed and reported HGF parameter estimates [31].” [referring to Suthaharan et al. (2021)].

The model in question here has 5 parameters (mu_02, omega2, kappa, mu_03, and omega3). While the results reported by Suthaharan et al are impressive, there has been no parameter recovery conducted in that paper either. Admittedly, Suthaharan et al. use a model that is very similar (same parameters apart from mu_02) to a model used in a previously published study by Reed et al. (2020). Reed et al report that parameter recovery for their 4-parameter model was 0.7 for omega2 and < 0.3 for kappa, mu_03 and omega 3.

Without a parameter recovery conducted in the data set of Suthaharan et al., we are likely justified to assume that the recovery of a 5-parameter model is not better.

I appreciate the fact that the aim of this paper was not to improve the HGF model, but rather to use an innovative approach to analyse how the already fitted model parameters relate to each other and to the OCD and paranoia scales. However, poor parameter recovery limits the degree to which parameters can be taken at face value to describe distinct aspects of the belief updating process.

Beyond improving the model (or showing good parameter recovery), this can be solved with a more careful (and toned down) interpretation of the results, a transparent reporting of the existing parameter recovery (or the lack of), and/or a discussion paragraph describing the limitations of models with limited parameter recovery scores.

In my opinion, the novel use of Bayesian graph models and the behavioural findings will be interesting to many readers of CP as is. I am simply not convinced that the parameters of the model “explain” the behavioral findings.

Minor issues:

The authors write:

“Excessive win-switch behavior (selecting a different choice following a rewarded outcome) may be a marker of oversampling, sensitivity to noisy sensory input, or overestimation of transition uncertainty.”

One simpler and more parsimonious explanation that can also be mentioned is reduced attention during the task, and a lack of task comprehension. As discussed before, the authors could directly address this with an inclusion of a noise parameter; or by adding this as a possible explanation and finding arguments for why this is not the case.

The authors further write:

“Conversely, lose-stay behavior (repeating a choice following an unrewarded outcome) becomes perseverative if sustained and may reflect an insensitivity to actual change, perhaps due to reduced learning about stimulus-action-outcome associations [22].”

As shown in Reed at al, Figure 6, none of the 4 parameters presented in the model capture the lose-stay behavior. This implies that the model used likely cannot capture the computational processes underlying lose-stay behavior, such as “reduced learning about stimulus-action-outcome associations”.

# Reviewer report: Round 2, Author Response

## Editorial Revision Requests:

Please address the remaining points in a final minor revision.

In brief, Reviewer 1 requests :

- a few clarifications and corrections, including checking a likely typo in Figure 5
- clarifying the terminology used to describe the HGF
- briefly commenting on the mix of Bayesian and frequentist methods
- adding support for claims about the advantages of the modelling approach
- noting the possible implications of the strong correlation between omega3 and kappa, including any relevant comments on identifiability or prior parameter recovery work.

Reviewer 2 is broadly supportive of publication, but asks:

- the interpretation of the HGF parameters be made more cautious
  - clearly acknowledge the limits imposed by the absence or weakness of parameter recovery evidence,
  - avoid overstating the extent to which the model parameters explain the behavioural findings
  - add a short discussion of this as a limitation
  - briefly considering simpler alternative explanations for excessive win-switching, such as reduced attention or task misunderstanding
  - revising the interpretation of lose-stay behaviour in light of the model’s apparent inability to capture that process

Apart from revising your manuscript based on review feedback, we also ask you to write a response to reviewers in which you explain how you have taken into account the reviewers’ comments.

## Reviewer A:

Recommendation: Accept Submission (No Revisions Necessary)

**Comments/Explanation (required). Please include references to any minor or major revisions required, as well as addressing any issues with language clarity. Authors will see these comments:**

Review of Excessive switching in OCD and paranoia arises from different deficits in belief-updating (Round 2)

The authors have carefully incorporated or responded to the points made in my original review, and I am happy to recommend the article for publication. Below are some minor points which the authors may find useful for a final, minor revision:

I appreciate the now expanded description of BGGM, which is very helpful and interesting.

I appreciate that it has been clarified that the HGF parameters were not obtained in this study, but were rather estimates made in a previous experiment. This of course renders many of my previous comments largely irrelevant (such as about parameter recovery and other quality checks of the parameter estimates). I apologize of the confusion, to the degree that it was due to inattentiveness on my part.

In figure five, the omega parameters seem to have a superscript 0, which I expect is a typo.

## Author’s response

We appreciate the reviewer’s critical attention to detail and have revised Figure 5 with the correct omega parameter notation. Figure files and generation code have been updated in this manuscript’s GitHub repository to reflect this correction accordingly (https://github.com/CharlotteFree/excessiveSwitching).

## Reviewer A:

call the HGF a “Bayesian mind model”, or, in full, a “variational Bayesian mind predictive processing model of perception and learning”,

On line 168, the authors call the HGF a (hierarchical) Bayesian model, and on line 946, a “hierarchical computational approach”. This is accurate, but ambiguous, because the term “hierarchical Bayesian model” also refers to using hierarchical Bayesian methods for fitting models to data model (the BGGM is for example a Bayesian model too, although not hierarchical; a mixed-effects model in brms would also be a hierarchical Bayesian computational model), which can also be done with other types of cognitive models, such as reinforcement learning (see e.g. hBayesDM for examples of this). In order to disambiguate, the authors might call the HGF a “Bayesian mind model”, or, in full, a “variational Bayesian mind predictive processing model of perception and learning”, or something similar.

## Author’s response

We agree with the reviewer in terms of the distinction and need for less ambiguous terminology. To address this, we removed the sentence, “Hierarchical Gaussian Filter (HGF) is one computational approach to modeling choice behavior.”, and replaced it on lines 210-211, as follows:

“We modeled beliefs using the Hierarchical Gaussian Filter (HGF), a Bayesian model for perception, learning, and action (Mathys et al., 2011).”

## Reviewer A:

The authors use an advanced Bayesian network or regressions, but also use a frequentist lme4 method earlier in the article. It makes one wonder why the authors didn't use Bayesian methods all the way through the article. There is no immediate need to change this, but the authors may consider very briefly commenting on it.

## Author’s response

We thank the reviewer for this thoughtful observation. Our use of both frequentist and Bayesian approaches reflects the distinct inferential aims of different components of this analysis, rather than a preference for one statistical framework over another. Frequentist generalized linear models and mixed-effects models were used for confirmatory, hypothesis-driven parameter-level inference, where they provide a well-established framework for estimating effects in hierarchical behavioral data and allow direct comparison with prior computational psychiatry and behavioral literature. In contrast, Bayesian Gaussian graphical models addressed a different inferential objective - characterizing conditional dependency structure and quantifying uncertainty at the network level - where a Bayesian framework offers particular advantages not required for confirmatory regression analyses. We have added a brief clarification in the Methods to underscore this choice. See Statistics, lines 318-322:

“Different analyses addressed distinct inferential levels, and statistical frameworks were selected according to the research question. Frequentist generalized linear and mixed-effects models were used for confirmatory parameter-level inference in hierarchically structured behavioral data, whereas Bayesian Gaussian graphical models were used to characterize conditional dependence structure and quantify uncertainty at the network level.”

## Reviewer A:

In line 182, the authors claim that Bayesian mind models are “better equipped to capture dynamic trial-by-trial choices and behavioral changes in response to task volatility (e.g., contingency reversal events), and to infer latent processes that lead to distinct behavioral strategies (i.e., win-switching, lose-staying)”.

- They authors may considering supporting this with one or more references; I am sure there are or could in principle be hierarchical reinforcement learning models appropriate for volatile environments too (although of course the HGF is specifically made for this).

## Author’s response

Thank you for this suggestion. We have added a reference to line 117 (Bottemanne, H. Bayesian brain theory: computational neuroscience of belief. *Neuroscience* 566, 198-204 (2025). https://doi.org/10.1016/j.neuroscience.2024.12.003.)

We have revised lines 119-123 (see below) with evidence for application of HGF models under conditions of uncertainty and volatility and added additional references with replication across various studies and study populations.

“HGF modeling has been applied to choice behavior in PRL tasks in human participants, non-human primates, and rodents to infer latent processes that lead to distinct behavioral strategies. For example, win-switch behavior has consistently correlated with paranoid beliefs and HGF volatility estimates, and this association has been repeatedly replicated across studies (Reed et al., 2020; Sheffield et al., 2022, 2023; Suthaharan et al., 2024, 2026.”

## Reviewer A:

Finally, although the strong correlation between omega3 and kappa is not due to methodological choices that the authors have made (since they use precomputed parameters), they might still comment on the possibility that this strong correlation indicates unidentifiability between the parameters. If this is the case, the correlation found by the BGGM is not empirical, but a consequence of the original fitting procedure. I don't know if the original authors did parameter recovery studies or similar to test for this, but the authors might report whether it was done, or whether such unidentifiability are reported for similar HGF models and for similar datasets.

## Author’s response

Thank you for this comment. To clarify, we make no claims of a strong correlation between omega3 and kappa in this manuscript - rather we provide evidence in support of the contrary. In Figure 6, there is no direct line between omega3 and kappa; in other words, the relationship between omega3 and kappa is mediated by their associations with mu03 and omega2.

For further reference, see “Figure-6_bggm-summary.txt” for a complete breakdown of each pairwise association (condensed for brevity in the supplementary materials). For each node-to-node comparison in Figure 6, we see the strongest support for the hypothesis of no association between omega3 and kappa (*P*(H0) = 0.792).

Other than these points, I find all my comments appropriately addressed, and congratulate the authors on thorough and clear work.

## Reviewer B:

Recommendation: Resubmit for Review

**Comments/Explanation (required). Please include references to any minor or major revisions required, as well as addressing any issues with language clarity. Authors will see these comments:**

The authors did an impressive amount of work to improve the readability and comprehension of the manuscript. I find the results of the OCD and paranoia on win-shift behaviour compelling and worth publishing.

However, as I clarify below, my main concerns that relate to the quality of the model used (including an inverse temperature parameter and making sure that the model used is well defined) are not addressed.

The authors write the following in the response to the parameter recovery issue:

“The present study analyzed this publicly available and peer-reviewed dataset, including the previously computed and reported HGF parameter estimates [31].” [referring to Suthaharan et al. (2021)].

The model in question here has 5 parameters (mu_02, omega2, kappa, mu_03, and omega3). While the results reported by Suthaharan et al are impressive, there has been no parameter recovery conducted in that paper either. Admittedly, Suthaharan et al. use a model that is very similar (same parameters apart from mu_02) to a model used in a previously published study by (Reed et al., 2020).

Reed et al report that parameter recovery for their 4-parameter model was 0.7 for omega2 and < 0.3 for kappa, mu_03 and omega 3.

Without a parameter recovery conducted in the data set of Suthaharan et al., we are likely justified to assume that the recovery of a 5-parameter model is not better.

## Author’s response

As the reviewer keenly and correctly pointed out, Reed et al., 2020 reported a strong parameter recovery for Omega2 (*r* = 0.702) but poor model recovery for higher level parameters (Mu03 and Omega3, *r’s* < 0.15). Poor recovery implies that there is likely a better model of these data and this task; we acknowledge that poor recovery or lack of recovery would have been a significant limitation of this study.

In response, we obtained the raw choice data from Suthaharan et al., 2021 and conducted a full parameter recovery analysis using 10 independent simulations of the 5-parameter model. The mean recovery statistics were as follows: Mu02, *r* = 0.8; Mu03, *r* = 0.74; kappa, *r* = 0.79; Omega2, *r* = 0.87; Omega3, *r* = 0.45. These results confirm that parameter recovery was robust enough to indicate the 3-level HGF model is identifiable and that parameters were reliably recovered.

We have added the following correlation matrix of true versus mean recovered parameter values to the supplementary materials (Supplementary Figure 9).

**Supplemental Figure 9:** Confusion matrix showing correlations between fitted and recovered parameters. Parameter recovery was quantified by correlating mean recovered parameters (across 10 independent simulations) to the original fitted parameters across participants. Mu02 (*μ^0^_2_*), Mu03 (*μ^0^_3_*), Kappa2 (κ), Omega2 (*ɷ_2_*) had robust parameter recovery values (*r’s* ≥ 0.74). Omega3 (ɷ*_3_*) was less reliably recovered (*r* = 0.45). These results support the identifiability of the model parameters under this 3-level HGF configuration.

We address this in-text (see lines 242-248) and reference a recent study that demonstrated strong parameter recovery and model identifiability for this 3-choice PRL paradigm. (Suthaharan, P., Castiello, S., Ang, Y.-S., & Corlett, P. (2026). Self-Reflection Protects Behavior from Volatile Beliefs Linked to Paranoia. Computational Psychiatry, 10(1), pp. 18–34. DOI: https://doi.org/10.5334/cpsy.150)

“Parameter recovery was conducted by correlating true and recovered parameter estimates across participants. Recovery performance was averaged across 10 independent simulations. Recovery was strong for four of the five parameters (r ≥ 0.74) and moderate for ω_3_ (r = 0.45), consistent with known differences in parameter identifiability in hierarchal Bayesian models such as the HGF (Supplementary Figure 9) [34,40]. Overall, these results support model identifiability for group-level inference under this 3-level HGF configuration.”

## Reviewer B:

I appreciate the fact that the aim of this paper was not to improve the HGF model, but rather to use an innovative approach to analyse how the already fitted model parameters relate to each other and to the OCD and paranoia scales. However, poor parameter recovery limits the degree to which parameters can be taken at face value to describe distinct aspects of the belief updating process.

Beyond improving the model (or showing good parameter recovery), this can be solved with a more careful (and toned down) interpretation of the results, a transparent reporting of the existing parameter recovery (or the lack of), and/or a discussion paragraph describing the limitations of models with limited parameter recovery scores.

## Author’s response

Please see response above. We performed parameter recovery analyses and now provide evidence of strong to moderate parameter recovery. We have added the following to the discussion section to address the limitations of weak parameter recovery and the implications for interpretation of Omega3 results (see lines 470-478):

“Parameter recovery procedures indicated that the model is largely identifiable, with four parameters showing strong recovery and ω_3_ showing comparatively lower precision (*r* = 0.45). While this is consistent with known limitations in hierarchical Bayesian model inversion, it is worth noting that poorer recovery performance implies that there is likely a better model of these data and this task. Correlation between parameters might mean the same, implying that the behavioral effects are not uniquely specified. Of course, there are instances when mechanisms trade off - for example, if studying whether prediction errors drive attentional allocation, we might want to attend to highly predictable things (Mackintosh, 1975) or highly unpredictable ones (Pearce and Hall, 1980). Yet, in both cases, prediction error and learning rate are correlated – albeit in different directions.”

## Reviewer B:

In my opinion, the novel use of Bayesian graph models and the behavioural findings will be interesting to many readers of CP as is. I am simply not convinced that the parameters of the model “explain” the behavioral findings.

## Author’s response

Poor recovery implies that there is likely a better model of these data and this task. Please see above.

## Reviewer B:

Minor issues:

The authors write:

“Excessive win-switch behavior (selecting a different choice following a rewarded outcome) may be a marker of oversampling, sensitivity to noisy sensory input, or overestimation of transition uncertainty.”

One simpler and more parsimonious explanation that can also be mentioned is reduced attention during the task, and a lack of task comprehension. As discussed before, the authors could directly address this with an inclusion of a noise parameter; or by adding this as a possible explanation and finding arguments for why this is not the case.

## Author’s response

We agree with the reviewer that this sentence should be simplified, which further brought to our attention that win-switch and lose-stay behaviors should be defined in the introduction section, prior to discussing the implication of these choice selection strategies.

We have adapted that section (lines 74-83) to read as follows:

“On each trial, participants select a choice, the outcome (win or lose) is revealed, and the next trial begins when the next choice is presented. This produces four possible selection strategies on the subsequent trial: win-stay (repeat a choice that was rewarded), win-switch (select a different choice following a reward choice), lose-stay (repeat a choice that was not rewarded), or lose-switch (select a different choice following an unrewarded choice). When win-switch or lose-stay strategies are applied excessively, and throughout the duration of the task, these behaviors can be indicative of impaired response selection strategies and underlying neurocognitive deficits (Kalhan, S., Magnard, R., Zhang, Z. *et al.* 2026. Uncertainty and reward histories have distinct effects on decisions after wins and losses. *Sci Rep* 16, 6795.). For example, excessive win-switching may be a marker of oversampling, heighted sensitivity to sensory input, reduced attention, or overestimation of transition uncertainty.”

As the reviewer points out, a lack of task comprehension is a possible explanation for win-switch or lose-stay behaviors. However, we argue that is unlikely given all human participants complete 6 practice trials following task instructions to minimize this possibility. We address this by adding the following sentence to the PRL Task Methods section (lines 176-177), as follows:

“To ensure comprehension of task instructions and objective, participants complete 6 practice trials prior to advancing to the task start.”

## Reviewer B:

The authors further write:

“Conversely, lose-stay behavior (repeating a choice following an unrewarded outcome) becomes perseverative if sustained and may reflect an insensitivity to actual change, perhaps due to reduced learning about stimulus-action-outcome associations [22].“

As shown in Reed at al., Figure 6, none of the 4 parameters presented in the model capture the lose-stay behavior. This implies that the model used likely cannot capture the computational processes underlying lose-stay behavior, such as “reduced learning about stimulus-action-outcome associations“.

## Author’s response

We agree that this sentence requires revision, given its context in the introduction, and that our model parameters do not reflect “learning” per say. We have amended lines 82-84 as follows, and added an additional reference:

“Conversely, lose-stay behavior becomes perseverative if sustained and may reflect an insensitivity to actual change or low confidence in one’s choices thereby causing their repetition [24, (Robbins et al., 2019)].”

In response to the reviewer’s comment that the model does not capture the latent processes underlying lose-stay behavior, we acknowledge that prior work in both empirical human data and model-simulated data, using the same PRL paradigm, has shown no significant relationship between lose-stay and paranoia. On the other hand, win-switch behavior has been consistently correlated with paranoia and is a validated marker of belief-volatility linked to paranoia [11,28,33,34,39,40], and hence we center the discussion around excessive switching as opposed to perseverative behavior.

Per the reviewer’s request to clarify the limitations of our study interpretations, we have also added the following to the discussion section (lines 518-525):

“Ultimately, this is not the last word on the explanations of win-switching or lose-staying, and future work will finesse our accounts. For now, we demonstrate that choice behaviors and latent belief-updating processes can distinguish psychiatric-like symptom phenomenology, even when those phenomena are highly correlated.”
